# Phylogenomic Analysis and Dynamic Evolution of Chloroplast Genomes in Salicaceae

**DOI:** 10.3389/fpls.2017.01050

**Published:** 2017-06-20

**Authors:** Yuan Huang, Jun Wang, Yongping Yang, Chuanzhu Fan, Jiahui Chen

**Affiliations:** ^1^Key Laboratory for Plant Diversity and Biogeography of East Asia, Chinese Academy of SciencesKunming, China; ^2^School of Life Sciences, Yunnan Normal UniversityKunming, China; ^3^Department of Biological Sciences, Wayne State University, DetroitMI, United States; ^4^Institute of Tibetan Plateau Research at Kunming, Kunming Institute of Botany, Chinese Academy of SciencesKunming, China

**Keywords:** chloroplast genome, phylogenomics, phylogenetic incongruence, NUPT, evolution, organellar horizontal gene transfer, Salicaceae

## Abstract

Chloroplast genomes of plants are highly conserved in both gene order and gene content. Analysis of the whole chloroplast genome is known to provide much more informative DNA sites and thus generates high resolution for plant phylogenies. Here, we report the complete chloroplast genomes of three *Salix* species in family Salicaceae. Phylogeny of Salicaceae inferred from complete chloroplast genomes is generally consistent with previous studies but resolved with higher statistical support. Incongruences of phylogeny, however, are observed in genus *Populus*, which most likely results from homoplasy. By comparing three *Salix* chloroplast genomes with the published chloroplast genomes of other Salicaceae species, we demonstrate that the synteny and length of chloroplast genomes in Salicaceae are highly conserved but experienced dynamic evolution among species. We identify seven positively selected chloroplast genes in Salicaceae, which might be related to the adaptive evolution of Salicaceae species. Comparative chloroplast genome analysis within the family also indicates that some chloroplast genes are lost or became pseudogenes, infer that the chloroplast genes horizontally transferred to the nucleus genome. Based on the complete nucleus genome sequences from two Salicaceae species, we remarkably identify that the entire chloroplast genome is indeed transferred and integrated to the nucleus genome in the individual of the reference genome of *P. trichocarpa* at least once. This observation, along with presence of the large nuclear plastid DNA (NUPTs) and NUPTs-containing multiple chloroplast genes in their original order in the chloroplast genome, favors the DNA-mediated hypothesis of organelle to nucleus DNA transfer. Overall, the phylogenomic analysis using chloroplast complete genomes clearly elucidates the phylogeny of Salicaceae. The identification of positively selected chloroplast genes and dynamic chloroplast-to-nucleus gene transfers in Salicaceae provide resources to better understand the successful adaptation of Salicaceae species.

## Introduction

The chloroplast (cp) is the photosynthetic organelle that provides energy for plants and algae. It is believed that chloroplasts arose from endosymbiosis between a photosynthetic bacterium and non-photosynthetic host (Dyall et al., 2004). The chloroplast has its own genome, which is generally non-recombinant and uniparentally inherited (Birky, 1995). Chloroplast genes are involved in major functions, which include sugar synthesis, starch storage, the production of several amino acids, lipids, vitamins, and pigments. They are also involved in key sulfur and nitrogen metabolic pathways. In angiosperms, most cp genomes are composed of circular DNA molecules ranging from 120 to 160 kb in length and have a quadripartite organization consisting of two copies of inverted repeats (IRs) of about 20–28 kb in size. These IRs divide the rest of cp genome into an 80–90 kb Large Single Copy (LSC) region and a 16–27 kb Small Single Copy (SSC) region (Jansen et al., 2005). The gene content and order of cp genomes of angiosperms are generally conserved, which encode four rRNAs, 30 tRNAs, and about 80 unique proteins (Chumley et al., 2006).

Chloroplast-derived DNA sequences have been widely used for phylogenetic studies, and complete cp genome sequences could provide valuable data sets for resolving complex evolutionary relationships (Jansen et al., 2007; Moore et al., 2010). However, acquiring large coverage of cp genomes has typically been limited by conventional DNA sequencing technology. As Next-Generation Sequencing technologies have revolutionized DNA sequencing (Shendure and Ji, 2008), it is now more convenient to obtain complete cp genome sequences with low cost, to extend gene-based phylogenetics to genome-based phylogenomics, and to examine phylogeny and evolutionary events of plant species using complete entire cp genome sequences.

Salicaceae s.str consists of two genera (Ohashi, 2001): *Salix* with about 450–580 species (Fang et al., 1999; Argus et al., 2010), and *Populus* with about 30 species (Argus et al., 2010). Species of Salicaceae are widely distributed in the world, except in Oceania and Antarctica. They are mostly found in the Northern Template Zone and are one of the main groups of trees and shrubs in those areas (Skvortsov, 1999; Argus et al., 2010). Plants of Salicaceae are often grown for ornament, shelterbelts, timber, pulp, and specialty wood products. Some shrub species of *Salix* are deemed as most suitable for bioenergy crops (Karp and Shield, 2008).

Because of dioecious reproduction, simple flowers, common natural hybridization, and large intraspecific phenotypic variation, both the resolution of taxonomy and the systematics of Salicaceae based on morphology, especially *Salix*, have been extremely difficult (Skvortsov, 1999; Argus et al., 2010). Molecular methods (e.g., molecular marker techniques, molecular phylogenetics and DNA barcoding) provide effective information for taxonomy, species identification, and phylogenetics of Salicaceae. However, previous molecular systematic analyses revealed that the phylogeny of Salicaceae, based on single or a few genetic markers, succeeds in resolving relationships in generic or sub-generic levels, but limits or has almost no resolution in infra-subgeneric level, specifically in subgenera *Chameatia*-*Vetrix* clade (Leskinen and Alstrom-Rapaport, 1999; Hamzeh and Dayanandan, 2004; Chen et al., 2008; Hardig et al., 2010). Therefore, DNA markers with higher resolution for phylogenetic analysis of unresolved lineages remain to be examined in Salicaceae.

Here, we report the complete cp genome sequences of three *Salix* species and further integrate the 11 available cp genomes of Salicaceae. Hence, all of the main lineages of Salicaceae have their representative species present in this study. The questions that we addressed in this study are: (1) What are potential DNA markers in cp genomes that can be used for phylogenetic analysis of Salicaceae? (2) What is the phylogenetic relation of Salicaceae based on phylogenomic analysis of complete Salicaceae cp genomes? (3) What are the structures and contents of cp genomes in Salicaceae? and (4) What are the evolution and dynamics patterns of cp genomes revealed by examining evolution of cp genes and DNA horizontally transferring events from cp to nucleus?

## Materials and Methods

### Plant Materials

Three species, *S. tetrasperma*, *S. babylonica*, and *S. oreinoma*, representing two subgenera of the genus *Salix*, were sampled. We collected healthy, tender and fresh leaves from adult plants of target species. The voucher herbarium specimens for the three sampled *Salix* species are all deposited in Herbarium of Kunming Institute of Botany, Chinese Academy of Sciences (KUN) (Supplementary Table S1).

### Chloroplast DNA Extraction, Sequencing, Genome, Assembly, and PCR-Based Validation

Total DNA enriched with cp DNA was extracted from 200 g of fresh leaves according to the methods of Jansen et al. (2005) and Zhang et al. (2011). Purified DNA (5 mg) was fragmented and used to construct short-insert libraries according to the manufacturer’s manual (Illumina Inc., San Diego, CA, United States). DNA from the different individuals was indexed by tags and pooled together in one lane of Illumina’s Genome Analyzer for sequencing.

We filtered out non-cp DNA reads from the raw sequences based on the known cp genome sequences. Next, the filtered reads were used to *de novo* assemble the cp genomes with SOAPdenovo software, which is specially designed to assemble Illumina short reads. SOAPdenovo pipeline (e.g., *k* = 31 bp and scaffolding contigs with a minimum size of 100 bp) can carry out accurate analyses of unexplored genomes, resolve repeat regions in contig assembly and improve gap closing, etc., in a cost effective way (Luo et al., 2012). Then, all contigs were mapped to the reference cp genome of *P. trichocarpa* using BLAST^1^ search from NCBI with default parameters. The orders of aligned contigs were determined according to the reference genome. Gaps between the *de novo* contigs were replaced with consensus sequences of raw reads mapped to the reference genomes.

Based on the reference genomes, the four junctions between LSC/IRs and SSC/IRs were confirmed with PCR-based product sequencing, respectively. To avoid assembly errors and to obtain high quality complete cp genome sequences, validation of assembly was also carried out with intensive PCR-based sequencing. We designed 39 pairs of primers (see Supplementary Table S2 for detail) based on the varied regions of the eight preliminary cp genome assemblies. PCR products were sequenced using the BigDye v3.1 Terminator Kit for ABI 3730xl (Life Technologies, Carlsbad, CA, United States). Sanger sequences and assembled genomes were aligned using Geneious 7 (Kearse et al., 2012) to determine if there were any differences. The complete cp genome sequences were deposited in GenBank (Supplementary Table S1).

### Chloroplast Genome Annotation

Genome annotation was accomplished using the Dual Organellar Genome Annotator (DOGMA) (Wyman et al., 2004) and also compared with the available complete chloroplast genome of *P. trichocarpa* (GenBank accession number NC009143) to annotate the genes encoding proteins, transfer RNAs (tRNAs), and ribosomal RNAs (rRNAs). All of the identified tRNA genes were further verified using the corresponding structures predicted by tRNAscan-Se v1.21 (Schattner et al., 2005).

### Phylogenetic and Network Analyses

We performed phylogenetic analyses based on different datasets, i.e., whole cp genome minus the second inverted repeat region (IRa) to avoid considering the same information twice, concatenated non-coding sequences (including intergenic spacers and introns), and concatenated exons of protein-coding genes (*ndhA* was excluded due to its pseudogenization in *S. babylonica*). Sequences were aligned with Muscle [62]. After manual correction in Geneious (Kearse et al., 2012), phylogenetic analyses were performed based on maximum likelihood (ML) criteria and Bayesian inference (BI). The ML analysis employing a GTR+G model of substitution for all datasets was run in RAxML v7.2.8 (Stamatakis, 2006), and a bootstrap analysis of 2000 replicates was performed simultaneously (option “-f a”). BI was performed with MrBayes v3.2 (Ronquist et al., 2012). Two independent Markov chain Monte Carlo (MCMC) chains were run, each with three heated and one cold chain for three million generations. Each chain started with a random tree, default priors, and sampling trees every 100 generations. The GTR+G model was used for all datasets as suggested by RaxML manual.

Topological incongruence among conflicting datasets was tested using Dendroscope v. 3.2.8 under the galled network consensus algorithm (Huson and Scornavacca, 2012). ML trees of concatenated protein coding sequences and whole genome sequence were used to infer phylogenetic networks.

### Molecular Evolution Analysis

We collected the coding DNA sequence (CDS) of each orthologous gene in eight *Populus* species and six *Salix* species, and aligned them with translation aligner using Geneious (Kearse et al., 2012), which could take into account frame shifts and premature stop codons, and generate codon-based alignments. We used the branch site model of PAML to compute the *K*a/*K*s ratio for orthologous genes in each external and internal branch of phylogeny trees that were generated based on protein coding sequence alignment with ML method. We tested two branch site models (with the parameters model = 2 and NSsites = 2): the “model 1” with both the branch site specific *K*a/*K*s and background *K*a/*K*s varying freely, and the “model 2” with the branch site specific *K*a/*K*s fixed at 1 and background *K*a/*K*s varying freely (Yang, 2007). We then performed the Likelihood Ratio Test (LRT), which tests whether the likelihood of the “model 1” is significantly different from that of the “model 2” by comparing two times the log likelihood difference. We computed *p*-values using a chi-square distribution with one degree of freedom (Yang, 1998).

### Analysis of Chloroplast-Nuclear DNA Transfer

We used the pipelines developed by UCSC genome browser to search for the homologous regions of the chloroplast genome in the nuclear genome for *P. trichocarpa*^2^ and *S. purpurea*^3^, respectively, since the nuclear genome sequences are only available for these two species (Tuskan et al., 2006). We first aligned the chloroplast genome to the nuclear genome of the two reference genomes with LASTZ (Harris, 2007). We then transformed the “lav” output format of LASTZ to “axt” format using lavToAxt. Finally we chained the “axt” files using axtChain and generated chain format outputs (Kent et al., 2003; Schwartz et al., 2003).

Although we did not set the identity filter of LASTZ for blocks or HSPs (high-scoring segment pairs), we computed the identity of the final homologous sequence pairs generated by axtChain. The identity ranges from 60 to 99%. Based on the chain file, we generated the homologous regions between the chloroplast genome and nuclear genome, which could represent chloroplast-nuclear DNA transfer events.

### Monte Carlo Sampling Testing

To test the hypotheses of whole chloroplast genome horizontally transferring to nucleus in *P. trichocarpa*, we conducted 100,000 Monte Carlo sampling test, which is implemented by the comparison of the observed data with random samples generated in accordance with the hypothesis being tested. The significance of the Monte Carlo sampling test is determined by the rank of the test criterion of the observed data relative to the test criteria of the random samples composing the reference set (Hope, 1968). Specifically, we assumed the whole chloroplast genome was split into eight fragments, which were inferred from the alignment between the chloroplast genome and nuclear genome in species *P. trichocarpa*. For each simulation, we randomly sampled the insertion locations of the eight fragments in the whole *P. trichocarpa* genome. We then counted the number of simulations, in which the five fragments (i.g. the 1st, 3rd, 5th, 7th, and 8th fragments) were inserted into the same chromosome within the range of the length of chromosome 13 and were arranged according to their previous order in the chloroplast genome. Next, we divided this number by the number of simulations (i.g. 100,000). This mathematical derivative is treated as the *P*-value for inference of the testing hypothesis significance.

## Results

### Genome Assembly and PCR-Based Validation

Using the Illumina Hiseq 2000 system, we obtain 1,392,310, 3,779,094, and 4,595,286 bp paired-end clean reads (average read length 91 bp) for *S. babylonica*, *S. tetrasperma*, and *S. oreinoma*, respectively. We mapped these sequence reads to the reference cp genome of *S. purpurea* and achieved at least 1600× (1615× for *S. babylonica*, 4384× for *S. tetrasperma*, 5330× for *S. oreinoma*) coverage for cp genome. Based on *de novo* and reference-guide assembly, we obtain the complete cp genome for *S. tetrasperma*. The assemblies of the other two cp genomes contain seven to eight gaps, and we filled the gaps using PCR-based sequencing.

Four junction regions of cp genomes were validated using PCR-based sequencing for each cp genome, respectively. Furthermore, in order to overcome the errors of heterogeneous indels from homopolymeric repeats (Moore et al., 2006; Yang et al., 2010), we conducted PCR-based validation to correct the errors. We designed 18 pairs of primers based on the variable regions of alignments to validate these sequences in each cp genome (Supplementary Table S2). In total, we amplified and sequenced ∼ 152 kb from all the three *Salix* species. Then, we compared these sequences directly to the assembled genomes and we observed no nucleotide mismatches or indels/insertions. This result confirmed the reliability of assembled chloroplast genome sequences. Finally, we obtain the complete cp genome sequences of *S. babylonica*, *S. tetrasperma*, and *S. oreinoma.*

### Salicaceae Chloroplast Genome Structure and Content

**FIGURE 1:**
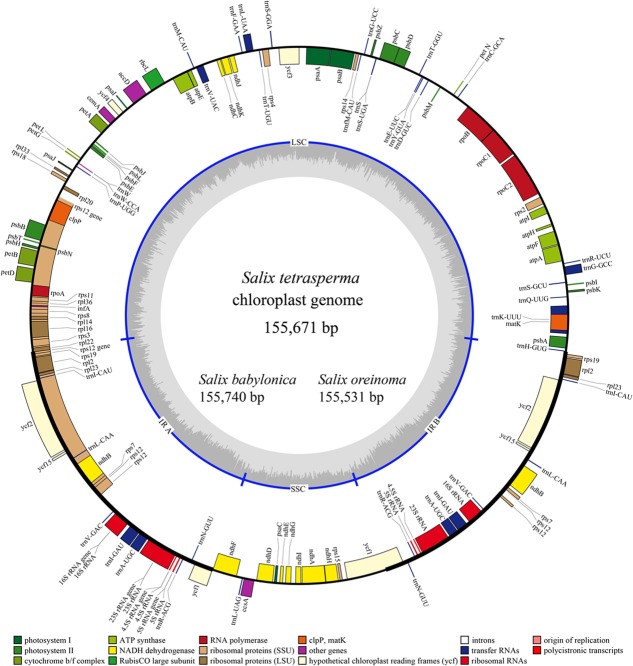
Gene map of the three sequenced *Salix* chloroplast genomes. Genes shown outside the outer circle are transcribed clockwise and those inside are transcribed counterclockwise. Genes belonging to different functional groups are color-coded. Dashed area in the inner circle indicates the GC content of the chloroplast genome of *Salix tetrasperma*.

The complete cp genomes of the three *Salix* species sequenced vary from 155,531 to 155,740 bp in size and exhibit a typical circular structure including a pair of IRs (range from 27384 to 27436 bp) and two single-copy regions (LSC, 84466–84580 bp; SSC, 15862–16323 bp). Each of the three cp genomes contains 111 unique genes (110 for *S. babylonica*) (**Figure 1** and **Table 1**), including 77 unique CDSs (76 for *S. babylonica* because of the pseudogenization of *ndhA*), four unique rRNAs, and 30 unique tRNAs. Seventeen genes contain introns; 14 of them (*atpF*, *ndhA*, *ndhB*, *petB*, *petD*, *rpl2*, *rpl16*, *rpoC1*, *trnA*^UGC^, *trnG*^GCC^, *trnI*^GAU^, *trnK*^UUU^, *trnL*^UAA^, and *trnV*^UAC^) exhibit one intron and three of them (*clpP*, *rps12*, and *ycf3*) contain two introns. All the CDSs have canonical ATG start codons except *ndhD*, which has GTG as the start codon.

**Table 1:** Features of chloroplast complete genomes in Salicaceae.

						Length of	Length of	Length of		No. of	No. of	No. of	No. of
	Genome	Length	Length	Length	Length	coding	protein coding	non-coding	GC	unique	unique	unique	unique
Species	size	of LSC	of SSC	of IRA	of IRB	sequence (%)	sequence (%)	sequence	(%)	gene	CDS	tRNA	rRNA
*Salix tetrasperma*^#^	155671	84584	16291	27398	27398	91593 (58.84)	80115 (51.47)	64078	36.7	113	77	30	4
*S. babylonica*^#^	155246	84516	15830	27450	27450	90483 (58.28)	79005 (50.89)	64763	36.6	112	76	30	4
*S. oreinoma*^#^	155531	84470	16213	27424	27424	92026 (59.17)	80187 (51.56)	63505	36.7	113	77	30	4
*S. interior*^∗^	156620	85980	16308	27166	27166	91738 (58.57)	80079 (51.13)	64882	37.0	113	77	30	4
*S. purpurea*^∗^	155590	84452	16220	27459	27459	92014 (59.14)	80175 (51.53)	63576	36.7	113	77	30	4
*S. suchowensis*^∗^	155214	84077	16221	27458	27458	92014 (59.28)	80175 (51.65)	63200	36.7	113	77	30	4
*Populus alba*^∗^	156505	84618	16567	27660	27660	92467 (59.08)	80628 (51.52)	64038	36.7	113	77	30	4
*P. balsamifera*^∗^	157094	84922	16499	27846	27827	92542 (58.91)	80703 (51.37)	64552	36.7	113	77	30	4
*P. euphratica*^∗^	156766	84887	16589	27644	27646	92406 (58.95)	80568 (51.39)	64360	36.7	113	77	30	4
*P. fremontii*^∗^	157446	85454	16318	27837	27837	92575 (58.80)	80736 (51.28)	64871	36.7	113	77	30	4
*P. tremula*^∗^	156067	84377	16490	27600	27600	92473 (59.25)	80634 (51.67)	63594	36.8	113	77	30	4
*P. yunnanensis*^∗^	155776	83955	16549	27636	27636	91404 (58.68)	79565 (51.08)	64372	36.8	113	77	30	4
*P. cathayana*^∗^	155449	83911	16488	27525	27525	92423 (59.46)	80584 (51.84)	63026	36.9	113	77	30	4
*P. trichocarpa*^∗^	157033	85129	16600	27652	27652	92440 (58.87)	80601 (51.33)	64593	36.7	113	77	30	4

*Sequence lengths are measured by bp. ^#^cp genome sequenced in this study. ^∗^cp genomes of Salicaceae are available in GenBank*.

### Molecular Marker Identification

**FIGURE 2:**
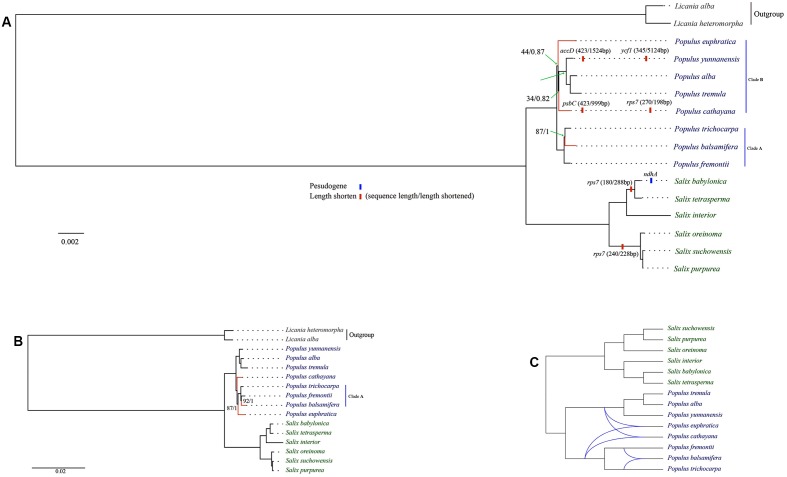
Maximum likelihood tree of Salicaceae. **(A)** The major variation events of protein coding sequences as compared to *Populus trichocarpa* and phylogeny based on concatenated protein coding dataset; **(B)** Phylogeny based on whole genome and concatenated non-coding datasets; **(C)** Rectangular cladogram of phylogenetic network. Clade supports were reported as maximum likelihood bootstrap support value/bayesian posterior probability (only those below100% or 1 were shown). Conflicting clades between different phylogenetic trees were highlighted by red.

**FIGURE 3:**
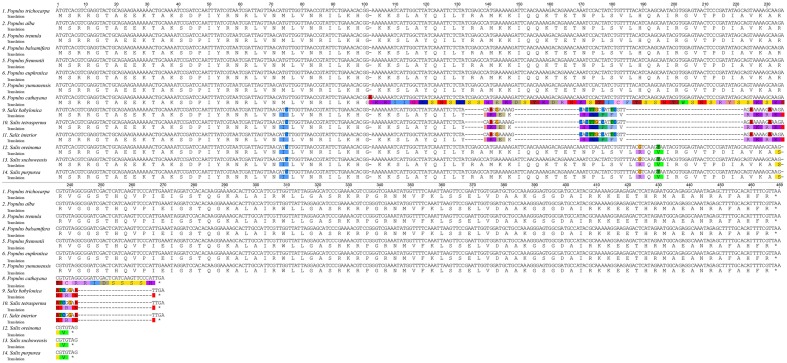
Alignment of translated *rps7* coding region in 14 Salicaceae species with *Licania* as outgroup.

Based on cp genome alignment from 14 Salicaceae species, we identified 30 highly divergent non-coding regions for implementation of phylogenetic analysis in *Salix*, *Populus* and Salicaceae. Those molecular markers are mostly derived from intergenic or intronic non-coding regions, though four protein-coding genes including *ccsA*, *rpl20*, *rps7* and *ndhA/ndhE* are also identified. Among the four genes, *rps7* is highly diverged and lineage-specific within genus *Salix*. The length of *rps7* is 468 bp for all *Populus* species except *P. cathayana*, whereas the three *Salix* species (i.g. *S. oreinoma*, *S. suchowensis*, and *S. purpurea*), which are closely related to subgenera *Chamaetia* and *Vetrix*, have identical *rps7* sequences with a length of 270 bp. The other three *Salix* species (i.g. *S. interior*, *S. babylonica*, and *S. tetrasperma*) in subgenus *Salix* have identical *rps7* sequences with a length of only 180 bp (**Figures 2A**, **3**, and Supplementary Table S3).

The phylogenetic resolution of the genus *Salix* is proven to be extremely difficult (Skvortsov, 1999; Argus et al., 2010; Wu et al., 2015) and no agreement on *Salix* phylogeny is made thus far. Molecular phylogenetic analysis in *Salix* only succeeds in resolving relationships within the subgeneric level, but fails in the taxonomic level under subgenus and shows almost no resolution in the clade with about 73% of *Salix* species (i.e., subgenera *Chamaetia* and *Vetrix*) (Leskinen and Alstrom-Rapaport, 1999; Azuma et al., 2000; Chen et al., 2010; Hardig et al., 2010; Abdollahzadeh et al., 2011; Wu et al., 2015). Therefore, there is a need to identify highly divergent regions in *Salix* cp genomes as molecular markers.

### Phylogenomic Analyses of Salicaceae

The genus *Populus* is comprised of ca. 30 species, which can be divided into six major clades (Eckenwalder, 1996; Argus et al., 2010). We selected eight species from four clades in our phylogenomic analyses. Our analysis indicates that *Populus* is monophyletic. Phylogeny, based on whole genome and concatenated non-coding datasets, shares the same topology (**Figure 2B**). However, phylogenetic relationship based on concatenated protein coding dataset is different in the topological positions of *P. euphratica*, *P. cathayana* and *P. balsamifera* (**Figure 2A**), as indicated by network analysis (**Figure 2C**). The three New World *Populus* species (*P. trichocarpa*, *P. balsamifera*, *P. fremontii*, Clade A in **Figure 2A**) form a robust monophyly. This clade is sister to the remaining *Populus* species in the protein coding gene tree (**Figure 2A**). However, *P. euphratica* is sister to *P. cathayana* from the tree based on whole cp genome and non-coding regions (**Figure 2B**). In the tree based on protein coding regions, *P. euphratica* and *P. cathayana* fall in the clade that contains all species from Old World with low support value and short branch length (clade B in **Figure 2A**). This topology is absent from previous studies with more *Populus* species sampled (Hamzeh and Dayanandan, 2004; Wang et al., 2014), indicating that insufficient sampling might have caused this topology.

### Molecular Evolution of Salicaceae Chloroplast Genome

We identified at least four possible pseudogenization events in *Salicaceae*: (1) *ndhA* in *S. babylonica*, which contains a 1039 bp deletion including a start codon in exon 1; (2) *accD* in *P. yunnanensis*, which loses 1056 bp (72%) of gene content; (3) *ycf1* in *P. yunnanensis*, which is deleted >5000 bp (94%) of DNA sequences; (4) *psbC* in *P. cathayana*, which misses ∼1000 bp (∼70%) of DNA sequences (**Figure 2A**).

We identified seven significantly positively selected genes (*atpE*, *rps7*, *ycf2*, *ccsA*, *petD*, *psbC*, and *psbJ*) that contain positively selected sites (**Table 2**). Among them, three positively selected genes (i.e., *rps7*, *petD*, and *psbC*) are identified in *P. cathayana*, *ycf2* is identified in *P. yunnanensis*, *ccsA* and *psbJ* are identified in *P. tremula*. An exception, *atpE*, shows significant positive selection on one site in the *Salix* subgenus *Salix* clade (i.e., *S. babylonica*, *S. tetrasperma*, and *S. interior*) (**Table 2**).

**Table 2:** Positive selection sites identified using Codeml under branch-site model.

Gene	Branch	Null	Alternative	*p*-value	Putative sites under positive selection, amino acid, and corresponding posterior probability
*atpE*	*S. babylonica, S. tetrasperma*, *S. interior*	-578.123	-566.714	1.78*E*-06	130 S 0.970^∗^
*rps7*	*P. cathayana*	-445.250	-437.826	1.17*E*-04	37 S 0.997^∗∗^; 38 L 0.992^∗∗^; 39 A 0.955^∗^; 40 Y 0.995^∗∗^; 41 Q 0.997^∗∗^; 42 I 0.909; 43 L 0.996^∗∗^; 44 Y 0.999^∗∗^; 45 R 0.986^∗^; 46 A 0.987^∗^; 47 M 0.961^∗^; 48 K 0.952^∗^; 49 K 0.989^∗^; 52 Q 0.978^∗^; 54 T 0.965^∗^
*ycf2*	*P. yunnanensis*	-9664.014	-9628.861	5.07*E*-17	2271 M 0.982^∗^; 2272 A 0.982^∗^; 2275 G 0.969^∗^
*ccsA*	*P. tremula*	-1585.549	-1581.119	2.92*E*-03	319 I 0.959^∗^
*petD*	*P. cathayana*	-499.511	-491.763	8.27*E*-05	116 N 0.988^∗^; 117 V 0.989^∗^
*psbC*	*P. cathayana*	-872.644	-829.745	1.99*E*-20	101 E 1.000^∗∗^; 102 V 1.000^∗∗^; 103 I 0.999^∗∗^; 104 D 0.997^∗∗^; 105 T 0.979^∗^; 106 F 0.998^∗∗^; 107 P 0.998^∗∗^; 108 Y 0.978^∗^; 109 F 0.974^∗^; 110 V 0.999^∗∗^; 111 S 1.000^∗∗^; 112 G 0.999^∗∗^; 113 V 1.000^∗∗^; 114 L 0.980^∗^; 115 H 1.000^∗∗^; 116 L 1.000^∗∗^; 117 I 0.974^∗^; 119 S 0.998^∗∗^; 120 A 1.000^∗∗^; 121 V 0.996^∗∗^; 122 L 0.995^∗∗^; 123 G 0.979^∗^; 124 F 0.999^∗∗^; 125 G 0.998^∗∗^; 127 I 0.978^∗^; 128 Y 0.998^∗∗^; 129 H 0.996^∗∗^; 130 A 1.000^∗∗^; 131 L 0.992^∗∗^; 132 L 0.996^∗∗^; 133 G 0.982^∗^; 134 P 0.980^∗^; 135 E 1.000^∗∗^; 136 T 0.997^∗∗^; 137 L 0.999^∗∗^; 138 E 0.998^∗∗^; 139 E 0.974^∗^
*psbJ*	*P. tremula*	-196.515	-192.834	6.66*E*-03	20 P 0.981^∗^

*^∗^Posterior probability > 95%, ^∗∗^Posterior probability > 99%. “Null” and “Alternative” columns list likelihood values obtained under the null model and alternative model.*

### Chloroplast-Nuclear DNA Transfer

**FIGURE 4:**
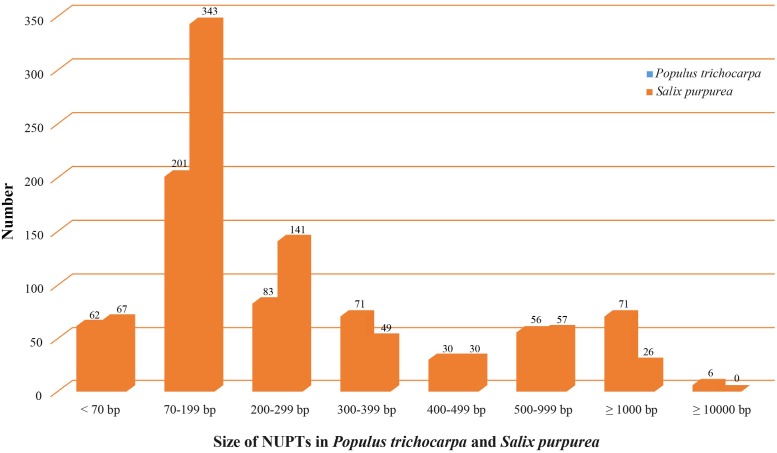
Illustration of NUPTs size distribution in *Populus trichocarpa* and *Salix purpurea*.

We used LASTZ (Harris, 2007) to search for NUPT including DNA fragments less than 200 bp. We identified 571 and 713 NUPTs with total ungapped lengths of ca. 536 and 193 kb in *P. trichocarpa* and *S. purpurea*, respectively (**Figure 4**, **Table 3**, and Supplementary Table S7). This result is different from a previous study that used BLAST method for searching NUPT (Yoshida et al., 2013). The number of NUPTs in *S. purpurea* is larger than that in *P. trichocarpa*, but the total length of NUPTs in *S. purpurea* is much lower than that in *P. trichocarpa* (**Table 3**).

**Table 3:** Sizes of chloroplast genome, of nuclear genomes, and of NUPTs detected by BlastZ.

Category	*Populus trichocarpa*	*Salix purpurea*
Nuclear genome size (Mb)	481	450
cp genome size (Kb)	157,033	155,590
Total number of NUPTs	574	713
Total length of NUPTs (bp)	2,869,374	491,553
Total gap length of NUPTs (bp)	2,333,068	298,700
Query aligned length (bp)	536,306	192,853
Query gap (bp)	1,204,611	565,676
Number of NUPTs genes in nuclear gene region	314	41
Number of NUPTs CDS in nuclear CDS regions	233	25
Length of cp CDS transferred in nuclear CDS region (bp)/proportion to NUPT length	126,219/23.5%	8,312/4.3%
Number of transferable genes	75	31
NUPTs in proportional of cp genome	341.50%	123.90%
NUPTs in proportional of nuclear genome	0.111%	0.043%
Number of transferable genes	75	31

*Number of NUPTs in *Populus trichocarpa* with length and identity no less than 100 bp and 90%, respectively, as is the criteria of Yoshida et al. (2013), is 175, and corresponding total length is 388,664bp. While in *Salix trichocarpa*, NUPT number in the criteria of Yoshida et al. (2013) is 141, and corresponding total length is 47,363bp*.

The fragmented assembly of *S. purpurea* genome (2015) might cause uncertainty for NUPT searching. Therefore, we subsequently discuss the plastid-to-nucleus DNA transfer in Salicaceae, based on the analysis from *P. trichocarpa*. The most abundant NUPTs in *P. trichocarpa* are around 70–199 bp (**Figure 4**). The number of NUPTs with 1000 bp longer is 77, and these large NUPTs are responsible for the majority of the total NUPT length (>75%, ca. 410 kb out of 536 kb). Furthermore, the average identity of those large NUPTs is 90.2%, and that of the six largest NUPTs with length greater than 10 kb is 99.4% (Supplementary Table S7), indicating that large NUPTs are relatively recently integrated into the nucleus (see Data Sheet S1 for detail). Additionally, all large NUPTs contain large gaps.

**FIGURE 5:**
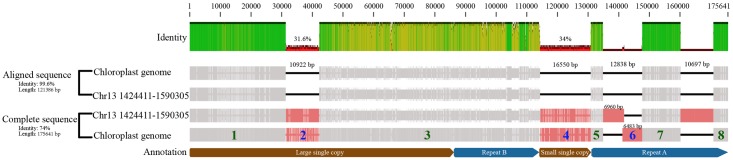
Illustration of whole chloroplast genome transferred to nucleus event in *Populus trichocarpa*. The “Aligned sequence” were the aligned chloroplast genome, and corresponding nuclear sequence (NUPT) which located in site 1,424,411 to 1,590,305 in chromosome 13 (i.e., fragment 1, 3, 5, 7, 8). And the “Complete sequence” also includes gaps. The fragments of the chloroplast genome that were separated by gaps were numbered, fragments with green number was present in nuclear chromosome, while the blue ones were not.

In our analyses, we identified five aligned regions (∼4,000–16,000 bp in length) and three/four gaps (∼7,000–15,000 bp in length) between the chloroplast and nuclear genomes in the same order as in the cp genome by LASTZ (**Figure 5**). We conducted MC sampling to demonstrate that it is more likely due to the insertion of the whole cp genome into the nuclear genome and generation of the gaps through insertion/translocation rather than the separate insertions of split cp genome fragments. Previously, it is also discovered that large cp fragments can be inserted into the nuclear genome based on the similarity between cp genome sequence and nuclear genome sequence using the sequence alignment tools (e.g., BLAST), and sequencing the insertion junction product (Yuan et al., 2002; Yoshida et al., 2013). Here, we used LASTZ as the alignment tool, which is a pairwise aligner for aligning DNA sequences and is originally designed to align the sequences in the size of human chromosomes and from different species (Harris, 2007). Therefore, it is more suitable to detect similarity in a large scale, such as between the cp genome and the nuclear genome. After alignment, we found that three large chunks of cp genomic regions are highly similar to three regions in nuclear genome, and more importantly, these regions are in the same order between the cp genome and nuclear genome.

Therefore, we conducted MC sampling to test the hypotheses of “whole cp genome insertion” or “split cp genome insertion,” which has not been done for detecting a whole cp genome transfer event before. We determine that the probability of those gaps produced by “split cp genome insertion” is less than 1*e*-05 and is significantly low by chance. Consequently, our simulation analysis supports that the whole chloroplast was transferred to the nuclear genome first and the gaps were created by subsequent insertion/translocation. Lastly, we found that the nucleus genes of NUPTs in *P. trichocarpa*, each of which matches multiple cp genes, contain multiple cp genes mostly in their original order in the cp genome (Supplementary Table S8). Taken together, all observations revealed by our analyses clearly favor the “bulk” and “insertion” DNA hypothesis of organelle-to-nucleus DNA horizontal transfer. Our approaches can serve as an example to analyze large-scale transfer/insertion events in other species.

## Discussion

### Highly Conserved cp Genomes of Salicaceae

We compared the three sequenced *Salix* cp genomes in this study with previous published complete cp genomes of 11 Salicaceae species, including eight *Populus* and three *Salix* species (Supplementary Table S2). We found that the structure and synteny of the cp genomes of the 14 Salicaceae species are highly conserved (Supplementary Figure S1). Also, the lengths of various segments, or parts (IRs, LSC, SSC, coding sequence and non-coding sequence), of the cp genomes are also quite conserved and vary in a small range (Supplementary Figure S2). We observed that four genes (i.g. *infA*, *rps16*, *rpl32*, and *ycf68*) were lost from Salicaceae cp genomes compared with other angiosperms, among which, *infA* and *rps32* were reported to be transferred into the nucleus (Millen et al., 2001; Ueda et al., 2007). The inverted repeat of the Salicaceae cp genome results in the complete duplication of *rps19*, *rpl2*, *rpl23*, *ycf2*, *ycf15*, *ndhB* and *rps7*, as well as exons 1 and 2 of *rps12*, all four rRNA genes (4.5S, 5S, 16S, and 23S) and seven tRNA genes (*trnI*^CAU^, *trnL*^CAA^, *trnV*^GAC^, *trnI*^GAU^, *trnA*^UGC^, *trnR*^ACG^, and *trnN*^GUU^). Both IRs of the Salicaceae cp genome run 1,695–1,907 bp into *ycf1*, which differs largely in genera *Salix* and *Populus*: 1,747 bp in all *Salix* species and 1,695–1,907 bp in *Populus* species, which result in most proportions of fluctuations in IR length. Additionally, IRb runs 50–51 bp into *rpl22* (Supplementary Figure S3).

Similar to other angiosperms, the IR (pairwise identity 98.1% for both IRa and IRb) regions are more conserved in the nine species than the LSC (pairwise identity 94.0%) and SSC (pairwise identity 94.9%) regions. Differences are observed in the cp genome in the 14 Salicaceae species including genome size, gene losses, the pseudogenization of protein-coding genes, and IR expansion and contraction (**Table 1** and Supplementary Figure S2).

### Phylogenomic Analyses of Salicaceae

Phylogenetic analysis, based on the complete cp genomes and non-coding and protein-coding datasets, indicates that the phylogeny of Salicaceae s.str, *Populus* and *Salix* s.l. are all resolved as monophyly, which is largely consistent with previous studies (Davis et al., 2005; Wurdack and Davis, 2009; Chen et al., 2010; Wang et al., 2014; Wu et al., 2015) (**Figure 2** and Supplementary Table S4). However, we identified the incongruence of phylogenies in Salicaceae that are inferred from these three datasets (i.e., whole genome and concatenated coding sequence between non-coding sequences of the cp genome). This incongruence might be caused by homoplasy, which could result from convergence, parallelism or reversal. Because the cp genome is inherited maternally as a single unit, the observed phylogenetic tree incongruences in our result are most unlikely caused by lineage sorting or hybridization/introgression, which are generally used to explain the conflict signals among characters of plant taxa including Salicaceae (Wang et al., 2014; Wu et al., 2015).

Our phylogenetic tree, based on the whole cp genome, coding-sequence and non-coding sequences of cp genome, shows that all conflicting branches are short (**Figures 2A,B**), which could be caused by fast species radiation or short stem-lineages (Wagele and Mayer, 2007). In this case, apomorphies evolved in stem-lineage may be rare, and chance similarities (non-phylogenetic signal produced by nucleotide substitution processes) that evolved later can accumulate and dominate in the form of phylogenetic-signal-like (i.e., false and misleading phylogenetic signals) patterns. It is difficult to distinguish these kinds of homoplasies from apomorphies, and consequently, this might lead to the wrong phylogenetic tree (Wagele and Mayer, 2007). Moreover, multiple nucleotide substitutions along long branches may destroy synapomorphies, resulting in accumulation of homoplasies along long branches and attracting distantly related clades to be clustered together in a topology (i.e., long-branch attraction) (Wagele and Mayer, 2007). Therefore, the conflict topologies in our phylogenomic trees most likely result from incomplete sampling or homoplasy. This is in line with previous cp DNA markers based on phylogenomic study of bamboo, which comes to the conclusion that homoplasy should be in account for conflicting phylogenetic signals between cp genome datasets (Ma et al., 2014). Moreover, our analyses show that the phylogenetic tree based on genomic data with large number of informative characters (e.g., cp genomes) should be used in caution when the examined taxa are quickly radiated or bear short branches.

Phylogenomic analysis indicates that the species of *Salix* were clustered in a robust monophyletic clade. Two robust subclades were resolved within *Salix*. One subclade includes the species of subgenera *Chametia* and *Vetrix*. Two species (*S. suchowensis* and *S. purpurea*) from subgenus *Vetrix* cluster as a monophyletic group, which is sister to a group including a species, *S. oreinoma* from subgenus *Chamaetia*. The other subclade is comprised of species from subgenus *Salix*, the New World species, *S. interior*, which is sister to a group containing two Old World species, *S. babylonica* and *S. tetrasperma* (**Figure 2**). The relationships resolved in this study are in line with previous phylogenetic studies of *Salix* (Azuma et al., 2000; Chen et al., 2010; Hardig et al., 2010; Wu et al., 2015), but we provide evidence from a phylogenomic perspective.

### Positively Selected Genes of Salicaceae Chloroplast Genomes

In the evolutionary process of a certain lineage of an organism, changing environments (e.g., climate changing) impose selective pressures and result in adaptive evolution. Identification of genes involved in this process (i.e., positively selected genes) is central to understanding the evolutionary pattern of organisms (Yang, 1998), and pinpointing specific targets for adaptive studies.

Among seven significantly positively selected genes in Salicaceae cp genomes, gene *rps7* shows a frame shift mutation. A single nucleotide insertion at site 102 causes a frame shift mutation for gene *rps7* and results in a large variation of an amino acid sequence that is shown under positive selection (**Figure 3**). This frame shift mutation further introduces an early stop codon and shortens its amino acid sequence about 42% of the length compared to other *Populus* species. The *rps7* gene encodes the ribosome S7 protein, also known as ribosomal protein S7 (uS7), which is crucial for the assembly and stability of the ribosome. S7 protein is an important part of the translation process, and is universally present in the small subunit of prokaryotic and eukaryotic ribosomes, and might play either a general or a specific regulatory role in translation initiation in the chloroplast (Fargo et al., 2001). The *rps7* gene of *P. cathayana* is positively selected, although it contains a premature stop codon caused by a frame shift mutation compared with other *Populus* species surveyed in this study (**Figure 3**). Despite the premature stop codon, this truncated *rps7* gene in *P. cathayana* has a conserved domain for the uS7 superfamily, as revealed by NCBI-CDD blast results (Supplementary Figure S4). This reveals that this shortened *rps7* gene may still function normally. Alternatively, even if the truncated cp rps7 cannot maintain its original function, it is possible that an intact copy of cp *rps7* has been transferred into the nucleus and can properly function in the nucleus genome. *P. cathayana* does not have complete genome sequences, so we searched the cp *rps7* gene in nucleus genome of *P. trichocarpa*, which has fine quality whole nuclear genome sequences. We observe that the Potri.013G138900.1 gene in chromosome 13 is identical to cp *rps7* in length and has 100% identity for both gene and coding sequences. This implies that *rps7* may have functionally transferred into the nucleus and the cp copy of *rps7* might be freely subject to natural selection.

Similarly, the protein coding sequences of *petD* and *psbC* genes in *P. cathayana* are shortened by about 29 and 70%, respectively. Compared to other Salicaceae species, both mutations are caused by a single nucleotide insertion. The *petD* gene encodes subunit IV of the cytochrome b_6_/f complex. It is required for photosynthetic electron transport and hence, supports photosynthetic growth. The mutations in the 5′ UTR or initiation codon can affect its function (Chen et al., 1993; Sakamoto et al., 1994; Sturm et al., 1994). The *psbC* gene encodes one of the components of the core complex of photosystem II. It binds chlorophyll and helps catalyze the primary light-induced photochemical processes of PSII (Rochaix et al., 1989; Cai et al., 2010). However, the effects of shortened coding sequences of *petD* and *psbC* on their function remains unknown, especially for *psbC*, which is shortened about 70% of the length. Our analysis finds that *petD* and *psbC* have been transferred into the coding region of the nuclear genome for three and eight times in *P. trichocarpa*, respectively (Supplementary Tables S5, S6).

Three of the last few amino acids of *ycf2* in *P. yunnanensis* are detected to be under significant positive selection. These amino acid changes are caused by a frame shift (a single nucleotide insertion) 6 bp ahead of them. Consequently, *ycf2* in *P. yunnanensis* is 21 bp shorter than in other Salicaceae species. The *ycf2* gene is a putative ATPase with unknown function. This gene exists in many plants, including non-photosynthetic plants. Previous experiments in tobacco indicate that it plays an essential role in cell survival in the tobacco chloroplast (Drescher et al., 2000). Our analysis indicates that *ycf2* is transferred into the nuclear genome 18 times and all targeted in protein-coding regions in *P. trichocarpa* (Supplementary Tables S5, S6).

We identified two positively selected genes, *ccsA* and *psbJ* in *P. tremula.* Species of *P. tremula* mainly distribute in cool temperate regions of Europe and Asia. The protein encoded by *ccsA* is a component of the cytochrome c synthase complex of the membrane-bound System II, and is required during the biogenesis of c-type cytochromes at the step of heme attachment (Xie et al., 1998). The *psbJ* encodes one of the components of the core complex of photosystem II. Experiments in tobacco indicate that plants with a mutated *psbJ* gene are unable to grow photoautotrophically (Hager et al., 2002). Our analysis shows that *ccsA* has been transferred to the coding region of the nuclear genome once, but *psbJ* is not transferred in *P. trichocarpa* (Supplementary Tables S5, S6).

All the positively selected genes locate in terminal species, except *atpE*, which shows significant positive selection on one site in the *Salix* subgenus, *Salix* clade (i.e., *S. babylonica*, *S. tetrasperma*, and *S. interior*) (**Table 2**). The *atpE* gene encodes the ε subunit of CF1 of the H^+^-translocating ATP synthase, and functions in part to prevent wasteful ATP hydrolysis by the enzyme (Cruz et al., 1997). The common ancestors of this clade might be adapted to temperate conditions and diverge in about early Oligocene (Wu et al., 2015). The positively selected *aptE* gene might be related to the adaptive evolution of the common ancestor group of *Salix* subgenus *Salix.*

As mentioned above, most of the positively selected protein-coding genes are transferred into the nucleus in *P. trichocarpa*. Positive selection is generally regarded as evidence of adaptive evolution, and these positive selected genes might have driven the successful adaptation of the selected taxa and/or lineages. However, the specific functions or effects of these positively selected cp genes in the target species remain unknown, and structural biological studies are needed to clarify the implication of these findings.

### Chloroplast-Nuclear DNA Transfer

Most of the chloroplast genes are transferred to nucleus and then deleted from the plastome. However, a transferred cp gene is not readily expressed in the nucleus, nor would it be able to give rise to a product equipped with the capability of returning to the chloroplast and ousting its progenitor chloroplast gene. The NUPT events have happened repeatedly and still are active during endosymbiotic evolution (Timmis et al., 2004; Cullis et al., 2009; Yoshida et al., 2013). Although some previous studies investigated the patterns of genomic integration of NUPTs in plant species, the mechanisms of cp-to-nucleus gene transfer are not well understood (Timmis et al., 2004; Lane, 2011; Yoshida et al., 2013).

The number of NUPTs we identified in *P. trichocarpa* is larger than that from a previous study (Yoshida et al., 2013), which used NCBI-BLASTN for NUPT identification instead of LASTZ, as in our study. However, the total length of NUPTs is similar. There is no clear explanation for the variation of the number and amount of NUPTs in plant species, but it may correlate with genome complexity, proportion of repetitive elements, and/or other factors (Yoshida et al., 2013).

Consistent with Yoshida et al. (2013), our analysis shows that large NUPTs are young. Therefore these large NUPTs may experience rapid recombination, insertion/deletion, and fragmentation; supporting previous results that the plant nuclear genome is in equilibrium between frequent integration and rapid elimination of plastid DNA (Matsuo et al., 2005).

There are two main hypotheses to explain the mechanism of DNA transferring from organelle-to-nucleus: the “bulk DNA” (DNA-mediated) view and “cDNA intermediate” (RNA-mediated) view (Timmis et al., 2004). Given the presence of large NUPTs containing both non-coding and coding regions, our results and most previous studies support “DNA-mediated” transfer hypothesis, (Matsuo et al., 2005; Michalovova et al., 2013; Yoshida et al., 2013). Despite the frequent occurrence of large NUPTs, e.g., nearly the entire chloroplast fragment in rice chromosome 10 (Yuan et al., 2002), whether the whole plastid genome can be transferred to the nucleus genome remains largely unknown or not specifically verified yet. We observe that the entire *P. trichocarpa* chloroplast genome is aligned to a region on chromosome 13 of the *P. trichocarpa* nuclear genome (Data Sheet S1). Together with MC sampling, our study clearly provide the evidence that the whole chloroplast genome horizontally transfers to the nuclear genome.

As discussed above, the whole chloroplast genome can be transferred to the nucleus, so the missing and pseudogenized genes in the Salicaceae chloroplast genome, e.g., *infA*, *ndhA*, *rpl16*, *rpl32*, could be transferred to the nucleus and may function properly. Similar cases have been reported previously, involving *rpl32* in *Populus* (Ueda et al., 2007) and *infA* in some angiosperm species (Millen et al., 2001). Our analyses and other studies show that organellar DNA horizontal transfer in plants is frequent. These transfers are thought to play an important role in gene and genome evolution in plants, and functional transfer of the chloroplast genes may facilitate the regulation of gene expression (Cullis et al., 2009; Yoshida et al., 2013). However, functional gene horizontal transferring from organelle-to-nucleus is rare as the transferred coding sequences must acquire gene promoter and terminator sequences for proper transcription in the nucleus, and must also acquire transit peptides necessary for reimporting the protein back into organelle (Timmis et al., 2004; Stegemann and Bock, 2006). Our result provides additional evidence of the organelle-to-nucleus horizontal functional gene transfer in species of *P. trichocarpa*, where the majority of cp coding sequences are transferred to the nucleus genome, and most of them remain as coding regions in the nucleus genome (Supplementary Tables S5, S6). However, further investigation is needed to determine whether these plastid-coding sequences in nuclear genome could function properly. Because chloroplast-to-nucleus transferred functional genes must acquire promoter and terminator sequences (Eckardt, 2006), it is necessary to identify the regulatory motifs of these chloroplast-to-nucleus transferred genes. Our discovery that large cp genome segments are transferred into the nuclear genome, implies that the transfer of the coding genes might be accompanied by the transfer of their promoter and terminator sequences, which could provide materials for those cp genes to properly function in the nuclear genome. However, further experiments (e.g., western bolt and genetic screen) and analyses are needed to confirm the function of the genes transferred from the chloroplast to the nucleus. Furthermore, the identification of the whole cp genome transfer event in *P. trichocarpa* is based on single genome analysis. It remains unclear whether this event is usual or even fixed in *P. trichocarpa*. Therefore, sequencing the genomes of more individuals is needed to further verify the whole cp genome transfer event in *P. trichocarpa.*

## Author Contributions

JC and YY designed research. YH and JC performed research. JC, JW, and YH analyzed data. JC, YH, JW, YY, and CF wrote the paper. All authors read and approved the final manuscript.

## Conflict of Interest Statement

The authors declare that the research was conducted in the absence of any commercial or financial relationships that could be construed as a potential conflict of interest.

## Footnotes

1 http://blast.ncbi.nlm.nih.gov/

2 https://phytozome.jgi.doe.gov/pz/portal.html#!info?alias=Org_Ptrichocarpa

3 https://phytozome.jgi.doe.gov/pz/portal.html#!info?alias=Org_Spurpurea

## References

[B1] Abdollahzadeh A. Osaloo S. K. Maassoumi A. (2011). Molecular phylogeny of the genus *Salix* (Salicaceae) with an emphasize to its species in Iran. *Iran. J. Bot.* 17 244–253.

[B2] Argus G. W. Eckenwalder J. E. Kiger R. W. Flora of North America. (2010). “Salicaceae,” in *Flora of North America*, ed. Flora of North America Editorial Committee (Oxford: Oxford University Press).

[B3] Azuma T. Kajita T. Yokoyama J. Ohashi H. (2000). Phylogenetic relationships of *Salix* (Salicaceae) based on rbcL sequence data. *Am. J. Bot.* 87 67–75. 10.2307/2656686 10636831

[B4] Birky C. W. (1995). Uniparental inheritance of mitochondrial and chloroplast genes - mechanisms and evolution. *Proc. Natl. Acad. Sci. U.S.A.* 92 11331–11338. 10.1073/pnas.92.25.11331 8524780 PMC40394

[B5] Cai W. H. Ma J. F. Chi W. Zou M. J. Guo J. K. Lu C. M. (2010). Cooperation of LPA3 and LPA2 is essential for photosystem II assembly in Arabidopsis. *Plant Physiol.* 154 109–120. 10.1104/pp.110.159558 20605914 PMC2938160

[B6] Chen J. H. Sun H. Wen J. Yang Y. P. (2010). Molecular phylogeny of *Salix* L. (Salicaceae) inferred from three chloroplast datasets and its systematic implications. *Taxon* 59 29–37.

[B7] Chen J. H. Sun H. Yang Y. P. (2008). Comparative morphology of leaf epidermis of *Salix* (Salicaceae) with special emphasis on sections Lindleyanae and Retusae. *Bot. J. Linn. Soc.* 157 311–322. 10.1111/j.1095-8339.2008.00809.x

[B8] Chen X. M. Kindle K. Stern D. (1993). Initiation codon mutations in the chlamydomonas chloroplast petD gene result in temperature-sensitive photosynthetic growth. *EMBO J.* 12 3627–3635. 8253086 10.1002/j.1460-2075.1993.tb06036.x PMC413638

[B9] Chumley T. W. Palmer J. D. Mower J. P. Fourcade H. M. Calie P. J. Boore J. L. (2006). The complete chloroplast genome sequence of *Pelargonium x hortorum*: organization and evolution of the largest and most highly rearranged chloroplast genome of land plants. *Mol. Biol. Evol.* 23 2175–2190. 10.1093/molbev/msl089 16916942

[B10] Cruz J. A. Radkowski C. A. McCarty R. E. (1997). Functional consequences of deletions of the N terminus of the epsilon subunit of the chloroplast ATP synthase. *Plant Physiol.* 113 1185–1192. 10.1104/pp.113.4.1185 12223668 PMC158241

[B11] Cullis C. A. Vorster B. J. Van Der Vyver C. Kunert K. J. (2009). Transfer of genetic material between the chloroplast and nucleus: how is it related to stress in plants? *Ann. Bot.* 103 625–633. 10.1093/aob/mcn173 18801916 PMC2707348

[B12] Davis C. C. Webb C. O. Wurdack K. J. Jaramillo C. A. Donoghue M. J. (2005). Explosive radiation of Malpighiales supports a mid-Cretaceous origin of modern tropical rain forests. *Am. Nat.* 165 E36–E65. 10.1086/428296 15729659

[B13] Drescher A. Ruf S. Calsa T. Carrer H. Bock R. (2000). The two largest chloroplast genome-encoded open reading frames of higher plants are essential genes. *Plant J.* 22 97–104. 10.1046/j.1365-313x.2000.00722.x 10792825

[B14] Dyall S. D. Brown M. T. Johnson P. J. (2004). Ancient invasions: from endosymbionts to organelles. *Science* 304 253–257. 10.1126/science.1094884 15073369

[B15] Eckardt N. A. (2006). Genomic hopscotch: gene transfer from plastid to nucleus. *Plant Cell* 18 2865–2867. 10.1105/tpc.106.049031

[B16] Eckenwalder J. E. (1996). “Systematics and evolution of *Populus*,” in *Biology of Populus and its Implications for Management and Conservation*, eds Stettler R. F. Bradshaw H. D. Heilman P. E. Hinckler T. M. (Ottawa, ON: Canadian Government Publishing), 7–32.

[B17] Fang Z. Zhao S. Skvortsov A. (1999). “Salicaceae,” in *Flora of China* Vol. 4 eds Wu Z. Y. Raven P. H. Hong D. Y. (Beijing: Science Press & Missouri Botanical Garden Press), 139–274.

[B18] Fargo D. C. Boynton J. E. Gillham N. W. (2001). Chloroplast ribosomal protein S7 of Chlamydomonas binds to chloroplast mRNA leader sequences and may be involved in translation initiation. *Plant Cell* 13 207–218. 10.1105/tpc.13.1.207 11158540 PMC102210

[B19] Hager M. Hermann M. Biehler A. Krieger-Liszkay A. Bock R. (2002). Lack of the small plastid-encoded PsbJ polypeptide results in a defective water-splitting apparatus of photosystem II, reduced photosystem I levels, and hypersensitivity to light. *J. Biol. Chem.* 277 14031–14039. 10.1074/jbc.M112053200 11827973

[B20] Hamzeh M. Dayanandan S. (2004). Phylogeny of *Populus* (Salicaceae) based on nucleotide sequences of chloroplast TRNT-TRNF region and nuclear rDNA. *Am. J. Bot.* 91 1398–1408. 10.3732/ajb.91.9.1398 21652373

[B21] Hardig T. Anttila C. Brunsfeld S. (2010). A phylogenetic analysis of *Salix* (Salicaceae) based on matK and ribosomal DNA sequence data. *J. Bot.* 2010:197696 10.1186/s12862-015-0311-7

[B22] Harris R. (2007). *Improved Pairwise Alignment of Genomic DNA.* Ph.D. thesis, Pennsylvania State University, State College, PA.

[B23] Hope A. C. A. (1968). A simplified Monte Carlo significance test procedure. *J. R. Stat. Soc. Ser. B* 30 582–598. 10.1088/0031-9155/54/3/005

[B24] Huson D. H. Scornavacca C. (2012). Dendroscope 3: an interactive tool for rooted phylogenetic trees and networks. *Syst. Biol.* 61 1061–1067. 10.1093/sysbio/sys062 22780991

[B25] Jansen R. K. Cai Z. Raubeson L. A. Daniell H. Depamphilis C. W. Leebens-Mack J. (2007). Analysis of 81 genes from 64 plastid genomes resolves relationships in angiosperms and identifies genome-scale evolutionary patterns. *Proc. Natl. Acad. Sci. U.S.A.* 104 19369–19374. 10.1073/pnas.0709121104 18048330 PMC2148296

[B26] Jansen R. K. Raubeson L. A. Boore J. L. dePamphilis C. W. Chumley T. W. Haberle R. C. (2005). Methods for obtaining and analyzing whole chloroplast genome sequences. *Methods Enzymol.* 395 348–384. 10.1016/S0076-6879(05)95020-9 15865976

[B27] Karp A. Shield I. (2008). Bioenergy from plants and the sustainable yield challenge. *New Phytol.* 179 15–32. 10.1111/j.1469-8137.2008.02432.x 18422906

[B28] Kearse M. Moir R. Wilson A. Stones-Havas S. Cheung M. Sturrock S. (2012). Geneious basic: an integrated and extendable desktop software platform for the organization and analysis of sequence data. *Bioinformatics* 28 1647–1649. 10.1093/bioinformatics/bts199 22543367 PMC3371832

[B29] Kent W. J. Baertsch R. Hinrichs A. Miller W. Haussler D. (2003). Evolution’s cauldron: duplication, deletion, and rearrangement in the mouse and human genomes. *Proc. Natl. Acad. Sci. U.S.A.* 100 11484–11489. 10.1073/pnas.1932072100 14500911 PMC208784

[B30] Lane N. (2011). Plastids, genomes, and the probability of gene transfer. *Genome Biol. Evol.* 3 372–374. 10.1093/gbe/evr003 21292628 PMC3101016

[B31] Leskinen E. Alstrom-Rapaport C. (1999). Molecular phylogeny of Salicaceae and closely related Flacourtiaceae: evidence from 5.8 S, ITS 1 and ITS 2 of the rDNA. *Plant Syst. Evol.* 215 209–227. 10.1007/BF00984656

[B32] Luo R. Liu B. Xie Y. Li Z. Huang W. Yuan J. (2012). SOAPdenovo2: an empirically improved memory-efficient short-read de novo assembler. *Gigascience* 1:18 10.1186/2047-217X-1-18 PMC3626529 23587118

[B33] Ma P. F. Zhang Y. X. Zeng C. X. Guo Z. H. Li D. Z. (2014). Chloroplast phylogenomic analyses resolve deep-level relationships of an intractable bamboo tribe Arundinarieae (poaceae). *Syst. Biol.* 63 933–950. 10.1093/sysbio/syu054 25092479

[B34] Matsuo M. Ito Y. Yamauchi R. Obokata J. (2005). The rice nuclear genome continuously integrates, shuffles, and eliminates the chloroplast genome to cause chloroplast-nuclear DNA flux. *Plant Cell* 17 665–675. 10.1105/tpc.104.027706 15705954 PMC1069690

[B35] Michalovova M. Vyskot B. Kejnovsky E. (2013). Analysis of plastid and mitochondrial DNA insertions in the nucleus (NUPTs and NUMTs) of six plant species: size, relative age and chromosomal localization. *Heredity* 111 314–320. 10.1038/hdy.2013.51 23715017 PMC3807264

[B36] Millen R. S. Olmstead R. G. Adams K. L. Palmer J. D. Lao N. T. Heggie L. (2001). Many parallel losses of infA from chloroplast DNA during angiosperm evolution with multiple independent transfers to the nucleus. *Plant Cell* 13 645–658. 10.1105/tpc.13.3.645 11251102 PMC135507

[B37] Moore M. J. Dhingra A. Soltis P. S. Shaw R. Farmerie W. G. Folta K. M. (2006). Rapid and accurate pyrosequencing of angiosperm plastid genomes. *BMC Plant Biol.* 6:17 10.1186/1471-2229-6-17 PMC1564139 16934154

[B38] Moore M. J. Soltis P. S. Bell C. D. Burleigh J. G. Soltis D. E. (2010). Phylogenetic analysis of 83 plastid genes further resolves the early diversification of eudicots. *Proc. Natl. Acad. Sci. U.S.A.* 107 4623–4628. 10.1073/pnas.0907801107 20176954 PMC2842043

[B39] Ohashi H. (2001). Salicaceae of Japan. *Sci. Rep. Tohoku Univ.* 40 269–396.

[B41] Rochaix J. D. Kuchka M. Mayfield S. Schirmerrahire M. Girardbascou J. Bennoun P. (1989). Nuclear and chloroplast mutations affect the synthesis or stability of the chloroplast psbc gene-product in *Chlamydomonas-reinhardtii*. *EMBO J.* 8 1013–1021. 2663467 10.1002/j.1460-2075.1989.tb03468.x PMC400908

[B42] Ronquist F. Teslenko M. van der Mark P. Ayres D. L. Darling A. Hohna S. (2012). MrBayes 3.2: efficient bayesian phylogenetic inference and model choice across a large model space. *Syst. Biol.* 61 539–542. 10.1093/sysbio/sys029 22357727 PMC3329765

[B43] Sakamoto W. Chen X. M. Kindle K. L. Stern D. B. (1994). Function of the *Chlamydomonas-reinhardtii* petD 5’ untranslated region in regulating the accumulation of subunit-IV of the cytochrome b_6/f_ Complex. *Plant J.* 6 503–512. 10.1046/j.1365-313X.1994.6040503.x 7987409

[B45] Schattner P. Brooks A. N. Lowe T. M. (2005). The tRNAscan-SE, snoscan and snoGPS web servers for the detection of tRNAs and snoRNAs. *Nucleic Acids Res.* 33 W686–W689. 10.1093/nar/gki366 15980563 PMC1160127

[B46] Schwartz S. Kent W. J. Smit A. Zhang Z. Baertsch R. Hardison R. C. (2003). Human-mouse alignments with BLASTZ. *Genome Res.* 13 103–107. 10.1101/gr.809403 12529312 PMC430961

[B47] Shendure J. Ji H. L. (2008). Next-generation DNA sequencing. *Nat. Biotechnol.* 26 1135–1145. 10.1038/nbt1486 18846087

[B48] Skvortsov A. K. (1999). *Willows of Russia and Adjacent Countries.* Joensuu: University of Joensuu.

[B49] Stamatakis A. (2006). RAxML-VI-HPC: maximum likelihood-based phylogenetic analyses with thousands of taxa and mixed models. *Bioinformatics* 22 2688–2690. 10.1093/bioinformatics/btl446 16928733

[B50] Stegemann S. Bock R. (2006). Experimental reconstruction of functional gene transfer from the tobacco plastid genome to the nucleus. *Plant Cell* 18 2869–2878. 10.1105/tpc.106.046466 17085684 PMC1693929

[B51] Sturm N. R. Kuras R. Buschlen S. Sakamoto W. Kindle K. L. Stern D. B. (1994). The petD gene is transcribed by functionally redundant promoters in *Chlamydomonas-reinhardtii* chloroplasts. *Mol. Cell. Biol.* 14 6171–6179. 10.1128/MCB.14.9.6171 8065350 PMC359144

[B52] Timmis J. N. Ayliffe M. A. Huang C. Y. Martin W. (2004). Endosymbiotic gene transfer: organelle genomes forge eukaryotic chromosomes. *Nat. Rev. Genet.* 5 123–U116. 10.1038/nrg1271 14735123

[B53] Tuskan G. A. DiFazio S. Jansson S. Bohlmann J. Grigoriev I. Hellsten U. (2006). The genome of black cottonwood, *Populus trichocarpa* (Torr. & Gray). *Science* 313 1596–1604. 10.1126/science.1128691 16973872

[B54] Ueda M. Fujimoto M. Arimura S. Murata J. Tsutsumi N. Kadowaki K. (2007). Loss of the rpl32 gene from the chloroplast genome and subsequent acquisition of a preexisting transit peptide within the nuclear gene in *Populus*. *Gene* 402 51–56. 10.1016/j.gene.2007.07.019 17728076

[B55] Wagele J. W. Mayer C. (2007). Visualizing differences in phylogenetic information content of alignments and distinction of three classes of long-branch effects. *BMC Evol. Biol.* 7:147 10.1186/1471-2148-7-147 PMC2040160 17725833

[B56] Wang Z. S. Du S. H. Dayanandan S. Wang D. S. Zeng Y. F. Zhang J. G. (2014). Phylogeny reconstruction and hybrid analysis of *Populus* (Salicaceae) based on nucleotide sequences of multiple single-copy nuclear genes and plastid fragments. *PLoS ONE* 9:e103645 10.1371/journal.pone.0103645 PMC4130529 25116432

[B57] Wu J. Nyman T. Wang D.-C. Argus G. W. Yang Y.-P. Chen J.-H. (2015). Phylogeny of *Salix* subgenus *Salix* s.l. (Salicaceae): delimitation, biogeography, and reticulate evolution. *BMC Evol. Biol.* 15:31 10.1186/s12862-015-0311-7 PMC4357182 25886526

[B58] Wurdack K. J. Davis C. C. (2009). Malpighiales phylogenetics: gaining ground on one of the most recalcitrant clades in the angiosperm tree of life. *Am. J. Bot.* 96 1551–1570. 10.3732/ajb.0800207 21628300

[B59] Wyman S. K. Jansen R. K. Boore J. L. (2004). Automatic annotation of organellar genomes with DOGMA. *Bioinformatics* 20 3252–3255. 10.1093/bioinformatics/bth352 15180927

[B60] Xie Z. Y. Culler D. Dreyfuss B. W. Kuras R. Wollman F. A. Girard-Bascou J. (1998). Genetic analysis of chloroplast c-type cytochrome assembly in *Chlamydomonas-reinhardtii*: one chloroplast locus and at least four nuclear loci are required for heme attachment. *Genetics* 148 681–692. 9504916 10.1093/genetics/148.2.681 PMC1459829

[B61] Yang M. Zhang X. W. Liu G. M. Yin Y. X. Chen K. F. Yun Q. Z. (2010). The complete chloroplast genome sequence of date palm (*Phoenix dactylifera* L.). *PLoS ONE* 5:e12762 10.1371/journal.pone.0012762 PMC2939885 20856810

[B62] Yang Z. H. (1998). Likelihood ratio tests for detecting positive selection and application to primate lysozyme evolution. *Mol. Biol. Evol.* 15 568–573. 10.1093/oxfordjournals.molbev.a025957 9580986

[B63] Yang Z. H. (2007). PAML 4: phylogenetic analysis by maximum likelihood. *Mol. Biol. Evol.* 24 1586–1591. 10.1093/Molbev/Msm088 17483113

[B64] Yoshida T. Furihata H. Y. Kawabe A. (2013). Patterns of genomic integration of nuclear chloroplast DNA fragments in plant species. *DNA Res.* 21 127–140. 10.1093/dnares/dst045 24170805 PMC3989485

[B65] Yuan Q. Hill J. Hsiao J. Moffat K. Ouyang S. Cheng Z. (2002). Genome sequencing of a 239-kb region of rice chromosome 10L reveals a high frequency of gene duplication and a large chloroplast DNA insertion. *Mol. Genet. Genomics* 267 713–720. 10.1007/s00438-002-0706-1 12207219

[B66] Zhang Y. J. Ma P. F. Li D. Z. (2011). High-throughput sequencing of six bamboo chloroplast genomes: phylogenetic implications for temperate woody bamboos (Poaceae: Bambusoideae). *PLoS ONE* 6:e20596 10.1371/journal.pone.0020596 PMC3105084 21655229

