# Supplementary material for: Phylogenomic Analysis and Dynamic Evolution of Chloroplast Genomes in Salicaceae
Source: Front Plant Sci. 2017 Jun 20;8:1050. doi: 10.3389/fpls.2017.01050 (PMC5476734; doi:10.3389/fpls.2017.01050)
Supplement: Supplementary file 2 [file Table_2.pdf]

Table S2 Primers used for gap closure

Name	Sequence
CJH_P1F	AGCCGCACTTAAAAGCCGAGT
CJH_P1R	CGTGGCCAAGCGGTAAGGCA
CJH_P2F	TCTTGGAGATTGTCTAATGCTTACTCTCA
CJH_P2R	TGCGCCGACGGATCCCTTAAC
CJH_P3F	TCTAGAAAGCGCGTTGTTTTGAATGC
CJH_P3R	TCAATCGGGAGACGTTTCGGCT
CJH_P4F	TCGAATCCCCGCTGCCTCCT
CJH_P4R	AAACTTTTCTGTGGAATGGAGGAAGGAAA
CJH_P5F	TGAGAGGGAGGGGTCAAACTTTCTT
CJH_P5R	GCATGTTTGTTGCCGGGGGC
CJH_P6F	CCACGAGGCGCTCAACGGAC
CJH_P6R	GCAGCGGTGTAGCATCAGATCCC
CJH_P7F	TGGTCGGGATAGCTCAGCAGGT
CJH_P7R	GCCGGAAAGTTGGATAGGGTGGC
CJH_P8F	TGAACTGCTTAGTTTTGTTTGCTGTGGT
CJH_P8R	ACGGTTCGAGTCCGTATAGCCCT
CJH_P9F	GGCAGTAGAGAATATTCGAGAGAACAGCG
CJH_P9R	ACTAGTTAGAGTGGGCATGAAGGAGC
CJH_P10F	ACCGTGTCAATTTCCAATTCTTGGT
CJH_P10R	CGCAGCTTGGTAGCGCGTTT
CJH_P11F	ACGAATAACGAAGGGGTTTTTCCTCCA
CJH_P11R	GGGATTTCCCCGTTCTCTACCCCG
CJH_P12F	CCATGTCCGCATTTCGTATCGAGGT
CJH_P12R	GAAGCAGGGCGTCGGGCAAT
CJH_P13F	ACTGCAGAACCGGACATGAGAGT
CJH_P13R	ACCAACTCATCGCTTCACATTATCTGG
CJH_P14F_LSC-IRb	TTTTATCACATAACTTCGTCCTCGAGCC
CJH_P14R_LSC-IRb	ACCAGAAAAGTTTGGGTAGAGCCGGA
CJH_P15F	ACTGTAGGGGAGGTCCTGCGG
CJH_P15R	TCCTTTGTTCCATAGTCCGGGGC
CJH_P16F_IRb-SSC	GTGGGCTCTATCGTGGAAAAGTCC
CJH_P16R_IRb-SSC	TGATTGGTCCTATAATCGTGGTTACATAGA
CJH_P17F	TGAGATTTTCACCTCATATGGCTCCTCG
CJH_P17R	TACGAGCTGCCGCTCAATCG
CJH_SSC-IRaF	TCTTCTCGTCTGTTTTTGCTCTCTGC
CJH_SSC-IRaR	CTGTTCACAACAGAAAAACTCTCCTCTGA
CJH_IRa-LSCF	CGTGGTCCCGGGCATCTACC
CJH_IRa-LSCR	TCCCTCTAGACCTAGCTGCTGTCG

Table S3 Most variable DNA markers in Salicaceae, *Populus* and *Salix* respectively.

**Salicaceae**			***Populus***			***Salix***		
**Marker**	**Aligned length (bp)**	**Segregate sites (percentage)**	**Marker**	**Aligned length (bp**	**Segregate sites (percentage)**	**Marker**	**Aligned length (bp)**	**Segregate sites (percentage)**
rps15-ycf1	454	55(12.1)	*trnS*^{GCU}*-trnG*^{GCC}	796	33(4.1)	*rps7*	240	21(8.8)
petA-psbJ	1038	100(9.6)	*trnH*^{GUG}*-psbA*	267	10(3.7)	*psbZ-trnG*^{UCC}	556	43(7.7)
rpl36-rps8	545	50(9.2)	*ndhC-trnV*^{UAC}	1423	43(3.0)	*rps15-ycf1*	431	33(7.7)
psbZ-trnG^{UCC}	693	61(8.8)	*rps15-ycf1*	439	12(2.7)	*trnG*^{GCC}*-trnR*^{UCU}	257	14(5.4)
psaC-ndhE	267	23(8.6)	*ycf4-cemA*	392	10(2.6)	*ndhF-trnL*^{UAG}	583	27(4.6)
trnG^{UCC}*-trnfM*^{CAU}	264	22(8.3)	*trnK*^{UUU}*-matK*	278	7(2.5)	*psaC-ndhE*	248	10(4.0)
psbK-psbI	488	38(7.8)	*trnR*^{ACG}*-trnN*^{GUU}	722	17(2.4)	*rpl36-rps8*	523	21(4.0)
trnH^{GUG}*-psbA*	278	21(7.6)	*rpl36-rps8*	516	12(2.3)	*psbC-trnS*^{UGA}	236	9(3.8)
ycf3-trnS^{GGA}	596	45(7.6)	*trnE*^{UUC}*-trnT*^{GGU}	260	6(2.3)	*ycf4-cemA*	392	14(3.6)
trnC^{GCA}*-petN*	416	31(7.6)	*trnF*^{GAA}*-ndhJ*	787	18(2.3)	*trnG*^{UCC}*-trnfM*^{CAU}	260	9(3.5)
trnL^{UAA}*-trnF*^{GAA}	380	28(7.4)	*trnC*^{GCA}*-petN*	395	9(2.3)	*psbK-psbI*	466	16(3.4)
trnM^{CAU}*-atpE*	276	20(7.2)	*rpl20*	354	8(2.3)	*ndhG-ndhI*	418	14(3.3)
trnG^{GCC}*-trnR*^{UCU}	309	22(7.1)	*rps2-rpoC2*	312	7(2.2)	*atpI-rps2*	215	7(3.3)
trnE^{UUC}*-trnT*^{GGU}	282	20(7.1)	*trnG*^{GCC}*-trnR*^{UCU}	268	6(2.2)	*trnL*^{UAA}*-trnF*^{GAA}	372	12(3.2)
rpoB-trnC^{GCA}	1425	100(7.0)	*trnM*^{CAU}*-atpE*	276	6(2.2)	*trnH*^{GUG}*-psbA*	261	8(3.1)
psbK-trnK^{UUU}	301	21(7.0)	*trnW*^{CCA}*-trnP*^{UGG}	248	5(2.0)	*ndhE-ndhG*	231	7(3.0)
ndhG-ndhI	432	30(6.9)	*psbZ-trnG*^{UCC}	696	13(1.9)	*trnK*^{UUU}*-matK*	269	8(3.0)
ndhE-ndhG	231	16(6.9)	*ndhG-ndhI*	432	8(1.9)	*rpl20*	354	10(2.8)
ndhF-trnL^{UAG}	684	47(6.97)	*psaJ-rpl33*	496	9(1.8)	*ccsA*	969	27(2.8)
trnS^{GCU}*-trnG*^{GCC}	802	55(6.9)	*psbM-trnD*^{GUC}	1277	23(1.8)	*trnS*^{GCU}*-trnG*^{GCC}	664	18(2.7)
ycf4-cemA	426	29(6.8)	*ndhF-trnL*^{UAG}	672	12(1.8)	*ycf3-trnS*^{GGA}	554	15(2.7)
trnS^{GGA}*-rps4*	293	19(6.5)	*rpoB-trnC*^{GCA}	1276	22(1.7)	*rpoB-trnC*^{GCA}	1276	34(2.7)
trnK^{UUU}*-matK*	278	18(6.5)	*trnS*^{GGA}*-rps4*	293	5(1.7)	*trnM*^{CAU}*-atpE*	270	7(2.6)
clpP-psbB	550	35(6.4)	*trnG*^{GCC} *intron*	704	12(1.7)	*psbE-petL*	1315	34(2.6)
psbC-trnS^{UGA}	236	15(6.4)	*ycf3-trnS*^{GGA}	592	10(1.7)	*atpH-atpI*	1021	26(2.5)
atpI-rps2	215	13(6.0)	*rpl16 intron*	1188	20(1.7)	*accD-psaI*	678	17(2.5)
ndhA intron	1197	68(5.9)	*petA-psbJ*	975	16(1.6)	*ndhF*	2274	57(2.5)
atpH-atpI	1068	58(5.4)	*ndhE*	306	5(1.6)	*ndhA intron*	1130	28(2.5)
rpl16 intron	1247	67(5.4)	*trnK*^{UUU}*-trnQ*^{UUG}	1298	21(1.6)	*trnC*^{GCA}*-petN*	407	10(2.5)
rpl20	354	19(5.4)	*petN-psbM*	1380	22(1.6)	*trnF*^{GAA}*-ndhJ*	783	19(2.4)

Table S4 Information of alignments used in phylogenetic analysis

	Whole genome	Whole genome minus ira	Noncoding	CDS
Aligned length	175956	148193	70945	67613
Variable sites	5900	5619	3889	1589
Informative sites	3569	3392	2341	995
Tree length	21846	20721	13582	6728
RI	0.961	0.961	0.958	0.967
CI	0.951	0.951	0.949	0.956

Note: Variable sites and informative sites were only caculated in ingroup taxon.

Table S5 Transferable CDSs and genes in *Populus trichocarpa* and *Salix purpurea*

		Populus trichocara		***Salix purpurea***	
**Un-transferable sequences in both species**	**transferable sequenecs in certain species**	**NO. transfered to gene of nucleus**	**NO. transfered to CDS of nucleus**	**NO. transfered to gene of nucleus**	**NO. transfered to CDS of nucleus**
4.5S_rRNA	*16S_rRNA*	3	-	-	-
5S_rRNA	*23S_rRNA*	4	-	1	-
atpI	*accD*	3	4	-	-
ccsA	*atpA*	4	4	1	1
matK	*atpB*	2	2	-	-
ndhC	*atpE*	4	3	4	2
ndhD	*atpF*	6	2	-	1
ndhE	*atpH*	6	5	-	-
ndhJ	*cemA*	2	2	-	-
petG	*clpP*	2	2	-	-
petL	*ndhA*	3	2	-	-
petN	*ndhB*	6	8	-	-
psaI	*ndhF*	6	6	3	4
psbZ	*ndhG*	3	3	-	-
rpl20	*ndhH*	2	2	-	-
rpl33	*ndhI*	2	1	1	-
rpl36	*ndhK*	2	2	-	-
rps14	*petA*	3	3	-	-
rps18	*petB*	8	6	-	-
trnF ^{GAA}	*petD*	4	3	1	1
trnG ^{GCC}	*psaA*	9	9	1	1
trnG ^{UCC}	*psaB*	3	3	1	1
trnH ^{GUG}	*psaC*	2	2	-	-
trnL ^{CAA}	*psaJ*	2	-	1	-
trnL ^{UAA}	*psbA*	7	5	2	-
trnL ^{UAG}	*psbB*	8	9	-	2
trnM ^{CAU}	*psbC*	8	8	-	-
trnR ^{ACG}	*psbD*	4	7	1	1
trnR ^{UCU}	*psbE*	3	3	1	-
trnS ^{GGA}	*psbF*	4	-	2	1
trnS ^{UGA}	*psbH*	10	7	2	-
trnT ^{GGU}	*psbI*	4	1	3	2
trnT ^{UGU}	*psbJ*	2	-	1	-
trnV ^{GAC}	*psbK*	4	4	1	-
trnV ^{UAC}	*psbL*	3	1	-	-
trnW ^{CCA}	*psbM*	2	2	3	-
	psbN	7	6	-	-
	psbT	6	1	-	-
	rbcL	3	5	-	-
	rpl14	3	3	-	-
	rpl16	2	2	-	-
	rpl2	8	8	-	1
	rpl22	2	2	-	-
	rpl23	4	1	1	-
	rpoA	3	2	1	1
	rpoB	3	4	-	-
	rpoC1	6	6	1	1
	rpoC2	8	8	-	-
	rps11	3	3	-	-

	*rps12**	7	-	1	-
	rps15	3	2	-	-
	rps19	7	3	-	-
	rps2	2	2	-	-
	rps3	7	4	-	-
	rps4	2	2	-	-
	rps7	8	8	-	-
	rps8	2	2	1	-
	trnA ^{UGC}	2	-	-	-
	trnC ^{GCA}	2	-	-	-
	trnD ^{GUC}	2	-	-	-
	trnE ^{UUC}	2	-	-	-
	trnfM ^{CAU}	2	-	-	-
	trnI ^{CAU}	2	-	-	-
	trnI ^{GAU}	3	-	1	-
	trnK ^{UUU}	4	-	1	-
	trnN ^{GUU}	2	-	-	-
	trnP ^{UGG}	4	-	-	-
	trnQ ^{UUG}	3	-	-	-
	trnS ^{GCU}	2	-	-	-
	trnY ^{GUA}	2	-	-	-
	ycf1	12	13	1	2
	ycf15	4	3	1	-
	ycf2	15	18	2	3
	ycf3	3	3	-	-
	ycf4	2	1	-	-
No. transferable		75	63	35	20
Total number		314	233	41	25
Total length (bp)		170550	112735	13090	7376

*We used the full length of *rps12* , which is ca. 30 kb in length.

Table S6 Tranfer of protein coding genes to nuclear genome in *Populus trichocarpa*

CP gene	CP gene length	CP gene start	CP gene end	NC gene ID	NC gene length	NC gene start	NC gene end	Identity score (%)	CP gene coverage	NC gene coverage
accD	1497	1002	1432	Potri.013G141100.1	363	0	363	93.65	0.29	1.00
accD	1497	1066	1423	Potri.005G150200.1	402	4	361	96.64	0.24	0.89
accD	1497	24	244	Potri.002G197100.1	222	0	217	87.92	0.15	0.98
accD	1497	4	162	Potri.013G141200.1	243	0	152	91.84	0.11	0.63
atpA	1524	0	1524	Potri.013G138000.1	1524	0	1524	100.00	1.00	1.00
atpA	1524	0	1524	Potri.005G154600.1	1443	0	1443	98.89	1.00	1.00
atpA	1524	16	1471	Potri.013G142800.1	1209	55	1209	98.01	0.95	0.95
atpA	1524	829	1014	Potri.012G059700.1	198	10	195	96.76	0.12	0.93
atpB	1497	55	1497	Potri.T005700.1	612	0	612	92.60	0.96	1.00
atpB	1497	1266	1497	Potri.002G211100.1	231	0	231	99.13	0.15	1.00
atpE	402	35	231	Potri.009G029700.1	231	38	231	93.26	0.49	0.84
atpE	402	52	251	Potri.007G054600.1	210	10	209	92.46	0.50	0.95
atpE	402	114	295	Potri.007G021100.1	216	27	216	90.06	0.45	0.88
atpF	555	144	555	Potri.013G137900.1	489	78	489	100.00	0.74	0.84
atpF	555	144	555	Potri.005G154700.1	489	78	489	100.00	0.74	0.84
atpH	246	0	246	Potri.005G154900.1	246	0	246	100.00	1.00	1.00
atpH	246	0	246	Potri.013G137700.1	246	0	246	100.00	1.00	1.00
atpH	246	0	246	Potri.017G074500.1	246	0	246	99.59	1.00	1.00
atpH	246	0	246	Potri.013G142700.1	279	34	279	98.78	1.00	0.88
atpH	246	0	246	Potri.019G028500.1	246	0	246	99.19	1.00	1.00
atpI	744	7	744	Potri.019G028600.1	741	4	741	98.91	0.99	0.99
ccsA	972	160	941	Potri.013G139400.1	447	0	447	93.05	0.80	1.00
cemA	687	45	687	Potri.016G094100.1	765	123	765	98.75	0.93	0.84
cemA	687	0	687	Potri.T058700.1	615	0	615	96.59	1.00	1.00
clpP	591	24	363	Potri.002G178800.1	357	0	345	89.44	0.57	0.97
clpP	591	165	591	Potri.017G140600.1	246	0	246	89.02	0.72	1.00
matK	1533	0	1533	Potri.013G143100.1	1518	0	1518	99.34	1.00	1.00
matK	1533	882	1533	Potri.013G138200.1	651	0	651	100.00	0.42	1.00
matK	1533	9	648	Potri.005G154400.1	618	40	618	97.58	0.42	0.94
matK	1533	1227	1384	Potri.017G027300.1	168	0	157	92.36	0.10	0.93
ndhA	1098	0	549	Potri.013G074800.1	573	0	548	95.23	0.50	0.96
ndhA	1098	667	1000	Potri.013G074900.1	336	4	336	97.29	0.30	0.99
ndhB	1539	16	1539	Potri.013G138800.1	1374	46	1374	98.49	0.99	0.97
ndhB	1539	168	1539	Potri.011G075100.1	1308	0	1308	97.96	0.89	1.00
ndhB	1539	918	1538	Potri.T074600.1	558	0	536	96.46	0.40	0.96
ndhB	1539	249	777	Potri.008G207100.1	663	0	528	96.97	0.34	0.80
ndhB	1539	249	777	Potri.008G208500.1	663	0	528	96.59	0.34	0.80
ndhB	1539	54	330	Potri.007G022800.1	366	0	276	96.38	0.18	0.75
ndhB	1539	1071	1250	Potri.007G084000.1	192	9	182	84.39	0.12	0.90
ndhB	1539	1382	1539	Potri.003G070700.1	234	77	234	91.08	0.10	0.67
ndhF	2289	57	915	Potri.T074500.1	702	0	642	95.33	0.37	0.91
ndhF	2289	771	1201	Potri.001G374900.1	390	0	382	92.76	0.19	0.98
ndhF	2289	1758	2085	Potri.009G010000.1	408	0	327	99.39	0.14	0.80
ndhF	2289	1593	1887	Potri.019G045000.1	300	0	294	97.96	0.13	0.98
ndhF	2289	0	269	Potri.013G139300.1	264	0	264	96.21	0.12	1.00
ndhF	2289	186	353	Potri.004G090300.1	165	0	165	95.15	0.07	1.00
ndhG	531	0	357	Potri.013G075100.1	357	0	357	96.92	0.67	1.00
ndhG	531	313	470	Potri.004G195500.1	279	121	279	96.18	0.30	0.57
ndhG	531	418	530	Potri.013G139500.1	252	0	103	87.37	0.21	0.41
ndhH	1182	30	1182	Potri.013G074700.1	786	0	786	94.90	0.97	1.00
ndhH	1182	0	910	Potri.013G139600.1	531	25	528	93.03	0.77	0.95
ndhI	531	130	531	Potri.013G075000.1	387	0	387	94.79	0.76	1.00
ndhK	678	285	560	Potri.018G044700.1	261	0	254	92.91	0.41	0.97
ndhK	678	90	615	Potri.013G141400.1	147	0	144	86.81	0.77	0.98
petA	963	0	963	Potri.016G094200.1	963	0	963	99.17	1.00	1.00
petA	963	0	963	Potri.T058600.1	915	0	915	97.92	1.00	1.00
petA	963	511	644	Potri.014G005100.1	156	1	134	87.22	0.14	0.85
petB	648	45	648	Potri.013G137300.1	645	42	645	99.50	0.93	0.93
petB	648	45	648	Potri.011G074700.1	645	42	645	98.34	0.93	0.93
petB	648	5	648	Potri.011G113500.1	558	14	558	97.06	0.99	0.97
petB	648	216	648	Potri.013G136700.1	432	0	432	100.00	0.67	1.00
petB	648	21	634	Potri.001G188100.1	330	0	316	86.08	0.95	0.96
petB	648	460	648	Potri.009G005000.1	189	1	189	97.34	0.29	0.99
petD	499	9	456	Potri.011G074600.1	459	51	459	96.81	0.90	0.89
petD	499	9	281	Potri.013G136800.1	321	51	321	98.15	0.55	0.84
petD	499	9	373	Potri.013G088900.1	426	51	384	91.33	0.73	0.78
psaA	2253	0	1318	Potri.016G089700.1	1137	0	1135	94.22	0.58	1.00
psaA	2253	436	1412	Potri.019G028100.1	1077	70	1046	99.69	0.43	0.91
psaA	2253	152	929	Potri.T007600.1	852	68	845	98.46	0.34	0.91
psaA	2253	0	726	Potri.003G065200.1	678	0	675	97.33	0.32	1.00
psaA	2253	0	630	Potri.013G141800.1	597	0	597	97.82	0.28	1.00
psaA	2253	34	528	Potri.007G087600.1	567	1	495	97.98	0.22	0.87
psaA	2253	1939	2253	Potri.002G040000.1	273	7	273	91.73	0.14	0.97
psaA	2253	2022	2253	Potri.004G127600.1	255	27	255	93.86	0.10	0.89
psaA	2253	287	381	Potri.005G150900.1	159	56	150	100.00	0.04	0.59
psaB	2205	4	1440	Potri.017G052700.1	831	16	831	86.01	0.65	0.98
psaB	2205	0	1199	Potri.009G016300.1	795	0	791	89.79	0.54	0.99
psaB	2205	1491	1672	Potri.003G066700.1	228	0	181	92.82	0.08	0.79
psaC	246	0	246	Potri.T027100.1	246	0	246	92.68	1.00	1.00
psaC	246	106	238	Potri.016G129800.1	216	109	216	85.05	0.54	0.50
psaI	114	0	114	Potri.016G094100.1	765	0	113	96.46	1.00	0.15
psbA	1062	0	1062	Potri.013G143200.1	1062	0	1062	100.00	1.00	1.00
psbA	1062	0	1062	Potri.013G138300.1	1062	0	1062	100.00	1.00	1.00

psbA	1062	619	1062	Potri.019G013500.1	342	4	342	86.57	0.42	0.99
psbA	1062	199	532	Potri.005G017900.1	312	0	291	81.94	0.31	0.93
psbA	1062	846	1062	Potri.019G013700.1	222	4	222	90.48	0.20	0.98
psbB	1527	0	1527	Potri.011G113900.1	1527	0	1527	100.00	1.00	1.00
psbB	1527	0	1527	Potri.013G137600.1	1527	0	1527	99.67	1.00	1.00
psbB	1527	0	1522	Potri.013G137100.1	1491	0	1491	99.13	1.00	1.00
psbB	1527	62	1527	Potri.017G140700.1	1197	5	1197	94.29	0.96	1.00
psbB	1527	440	740	Potri.010G093500.1	327	0	281	91.81	0.20	0.86
psbB	1527	1311	1527	Potri.004G188000.1	228	12	228	99.07	0.14	0.95
psbB	1527	735	1152	Potri.014G092700.1	201	18	201	83.14	0.27	0.91
psbB	1527	231	392	Potri.019G044300.1	270	0	152	86.18	0.11	0.56
psbB	1527	552	687	Potri.002G108200.1	165	0	135	90.37	0.09	0.82
psbC	1429	664	1425	Potri.014G188300.1	666	6	666	92.26	0.53	0.99
psbC	1429	198	828	Potri.008G208700.1	621	0	620	92.87	0.44	1.00
psbC	1429	114	827	Potri.019G047500.1	723	0	580	91.68	0.50	0.80
psbC	1429	198	728	Potri.008G207300.1	525	0	525	93.46	0.37	1.00
psbC	1429	880	1429	Potri.009G016500.1	513	0	513	90.51	0.38	1.00
psbC	1429	0	320	Potri.010G032700.1	360	42	360	94.97	0.22	0.88
psbC	1429	533	830	Potri.T020500.1	375	0	292	96.56	0.21	0.78
psbC	1429	355	685	Potri.019G024500.1	333	93	333	83.33	0.23	0.72
psbD	1062	59	1062	Potri.008G208600.1	660	5	660	92.37	0.94	0.99
psbD	1062	59	1062	Potri.008G207200.1	660	5	660	92.37	0.94	0.99
psbD	1062	18	269	Potri.001G331000.1	264	3	254	96.02	0.24	0.95
psbD	1062	602	720	Potri.001G457100.1	282	89	207	93.22	0.11	0.42
psbD	1062	611	722	Potri.006G141600.1	213	77	188	95.50	0.10	0.52
psbD	1062	612	720	Potri.017G082400.1	168	0	108	94.44	0.10	0.64
psbD	1062	602	699	Potri.013G081000.1	174	77	174	90.72	0.09	0.56
psbE	252	0	236	Potri.011G095300.1	252	0	236	90.25	0.94	0.94
psbE	252	0	122	Potri.013G141000.1	291	84	205	97.52	0.48	0.42
psbE	252	0	100	Potri.002G237300.1	246	0	100	94.00	0.40	0.41
psbH	222	0	222	Potri.011G113600.1	222	0	222	100.00	1.00	1.00
psbH	222	0	222	Potri.013G136900.1	222	0	222	100.00	1.00	1.00
psbH	222	0	222	Potri.011G074800.1	222	0	222	99.55	1.00	1.00
psbH	222	0	222	Potri.019G028200.1	222	0	222	99.10	1.00	1.00
psbH	222	0	222	Potri.013G137400.1	222	0	222	98.20	1.00	1.00
psbH	222	20	222	Potri.002G174000.1	207	5	207	96.04	0.91	0.98
psbH	222	3	217	Potri.009G079300.1	198	0	198	90.16	0.96	1.00
psbI	111	0	111	Potri.015G020100.1	111	0	111	94.59	1.00	1.00
psbK	195	0	195	Potri.005G154500.1	195	0	195	100.00	1.00	1.00
psbK	195	0	195	Potri.013G138100.1	195	0	195	100.00	1.00	1.00
psbK	195	0	195	Potri.013G143000.1	195	0	195	100.00	1.00	1.00
psbK	195	36	195	Potri.019G109500.1	336	177	336	91.19	0.82	0.47
psbL	117	0	113	Potri.002G237300.1	246	102	215	95.58	0.97	0.46
psbM	105	0	105	Potri.013G142100.1	105	0	105	100.00	1.00	1.00
psbM	105	0	105	Potri.012G101300.1	105	0	105	98.10	1.00	1.00
psbN	132	0	132	Potri.011G113700.1	132	0	132	100.00	1.00	1.00
psbN	132	0	132	Potri.013G137000.1	132	0	132	100.00	1.00	1.00
psbN	132	0	132	Potri.013G137500.1	132	0	132	100.00	1.00	1.00
psbN	132	0	132	Potri.019G028300.1	132	0	132	100.00	1.00	1.00
psbN	132	0	126	Potri.009G079200.1	153	0	126	96.83	0.95	0.82
psbN	132	0	126	Potri.009G004900.1	156	22	148	96.03	0.95	0.81
psbT	108	0	103	Potri.011G113800.1	102	0	102	97.06	0.95	1.00
psbZ	189	0	140	Potri.009G016400.1	141	0	140	95.71	0.74	0.99
rbcL	1437	0	399	Potri.T005800.1	477	21	420	96.24	0.28	0.84
rbcL	1437	1076	1420	Potri.012G062600.1	444	92	436	97.09	0.24	0.77
rbcL	1437	205	615	Potri.010G033200.1	342	1	342	87.06	0.29	1.00
rbcL	1437	304	554	Potri.T063100.1	414	94	344	98.40	0.17	0.60
rbcL	1437	1259	1423	Potri.T006000.1	207	38	202	97.56	0.11	0.79
rpl14	369	0	338	Potri.013G140600.1	336	0	336	98.51	0.92	1.00
rpl14	369	179	369	Potri.006G139000.1	201	11	201	95.26	0.51	0.95
rpl14	369	0	179	Potri.004G132800.1	192	0	179	90.50	0.49	0.93
rpl16	408	9	357	Potri.013G140500.1	411	66	408	96.49	0.85	0.83
rpl16	408	218	408	Potri.013G136600.1	450	260	450	100.00	0.47	0.42
rpl2	834	0	834	Potri.013G138600.1	837	0	837	99.76	1.00	1.00
rpl2	834	0	834	Potri.013G136500.1	726	0	726	97.66	1.00	1.00
rpl2	834	0	834	Potri.013G138400.1	720	0	720	97.78	1.00	1.00
rpl2	834	108	834	Potri.013G143300.1	735	0	735	99.31	0.87	1.00
rpl2	834	0	401	Potri.005G154300.1	402	0	395	98.21	0.48	0.98
rpl2	834	0	407	Potri.011G074400.1	525	0	402	95.97	0.49	0.77
rpl2	834	400	746	Potri.011G074300.1	369	0	346	99.13	0.41	0.94
rpl2	834	400	608	Potri.018G048200.1	309	21	229	88.46	0.25	0.67
rpl22	399	0	262	Potri.013G136600.1	450	0	262	100.00	0.66	0.58
rpl22	399	226	399	Potri.013G140300.1	282	64	235	97.08	0.43	0.61
rpl23	282	85	282	Potri.004G104100.1	234	37	234	95.43	0.70	0.84
rpl33	201	0	201	Potri.003G067400.1	201	0	201	100.00	1.00	1.00
rpoA	1029	0	973	Potri.013G140800.1	363	0	363	91.97	0.95	1.00
rpoA	1029	143	274	Potri.016G064900.1	150	5	136	89.31	0.13	0.87
rpoB	3213	0	3213	Potri.013G142200.1	3018	0	3018	99.37	1.00	1.00
rpoB	3213	1787	2514	Potri.006G133600.1	339	26	330	83.52	0.23	0.90
rpoB	3213	2101	2267	Potri.004G065700.1	270	71	229	85.81	0.05	0.59
rpoB	3213	1073	1183	Potri.006G199700.1	225	107	216	90.83	0.03	0.48
rpoC1	2070	882	1822	Potri.019G028000.1	939	0	939	98.94	0.45	1.00
rpoC1	2070	21	754	Potri.013G142300.1	720	0	720	98.47	0.35	1.00
rpoC1	2070	1053	1951	Potri.013G142400.1	1431	0	643	97.05	0.43	0.45
rpoC1	2070	21	453	Potri.019G027900.1	438	0	432	99.54	0.21	0.99
rpoC1	2070	1401	1759	Potri.001G086200.1	375	0	361	88.95	0.17	0.96

rpoC1	2070	706	889	Potri.002G106700.1	237	31	217	93.44	0.09	0.78
rpoC2	4227	0	1546	Potri.003G067300.1	1527	12	1527	98.02	0.37	0.99
rpoC2	4227	1549	3206	Potri.019G027800.1	1416	0	1415	98.16	0.39	1.00
rpoC2	4227	43	1053	Potri.013G142400.1	1431	643	1431	97.21	0.24	0.55
rpoC2	4227	3290	3696	Potri.009G133700.1	405	0	405	96.21	0.10	1.00
rpoC2	4227	699	964	Potri.019G027700.1	273	0	265	99.25	0.06	0.97
rpoC2	4227	3428	3635	Potri.013G142500.1	207	0	207	100.00	0.05	1.00
rpoC2	4227	4020	4227	Potri.017G063100.1	207	0	207	100.00	0.05	1.00
rpoC2	4227	6	133	Potri.007G060700.1	165	18	145	95.28	0.03	0.77
rps11	417	158	417	Potri.010G032600.1	333	74	333	94.59	0.62	0.78
rps11	417	159	417	Potri.019G046800.1	258	0	258	94.19	0.62	1.00
rps11	417	167	417	Potri.010G033100.1	258	8	258	94.80	0.60	0.97
rps12	372	114	345	Potri.011G074500.1	306	63	294	99.57	0.62	0.75
rps12	372	159	345	Potri.013G139000.1	231	34	219	98.38	0.50	0.80
rps12	372	148	345	Potri.004G127900.1	225	16	213	95.94	0.53	0.88
rps12	372	161	344	Potri.008G208300.1	258	62	245	93.99	0.49	0.71
rps12	372	168	345	Potri.017G060900.1	189	0	177	87.01	0.48	0.94
rps12	372	0	114	Potri.004G092700.1	129	0	114	87.72	0.31	0.88
rps15	273	0	273	Potri.012G027700.1	273	0	273	99.27	1.00	1.00
rps15	273	40	273	Potri.001G116400.1	270	37	270	99.57	0.85	0.86
rps19	280	0	198	Potri.011G074200.1	777	0	198	98.99	0.71	0.25
rps19	280	32	198	Potri.019G013600.1	204	38	204	90.91	0.59	0.81
rps19	280	129	280	Potri.019G013400.1	150	0	150	92.62	0.54	1.00
rps2	711	90	711	Potri.019G028700.1	621	0	621	99.36	0.87	1.00
rps2	711	0	711	Potri.013G142600.1	498	0	498	96.18	1.00	1.00
rps3	657	237	657	Potri.004G192200.1	399	0	399	92.53	0.64	1.00
rps3	657	0	288	Potri.012G047500.1	288	0	288	97.92	0.44	1.00
rps3	657	315	539	Potri.013G140400.1	219	0	219	95.43	0.34	1.00
rps3	657	432	657	Potri.002G235600.1	225	0	225	95.56	0.34	1.00
rps4	606	149	559	Potri.013G141500.1	369	0	369	95.39	0.68	1.00
rps4	606	186	300	Potri.019G042400.1	114	0	114	89.47	0.19	1.00
rps7	481	0	481	Potri.013G138900.1	468	0	468	97.63	1.00	1.00
rps7	481	72	459	Potri.013G140200.1	288	0	288	91.04	0.80	1.00
rps7	481	0	475	Potri.008G208400.1	264	0	258	89.15	0.99	0.98
rps7	481	50	335	Potri.004G128000.1	261	0	261	93.03	0.59	1.00
rps7	481	79	315	Potri.T074700.1	222	0	222	94.44	0.49	1.00
rps7	481	213	481	Potri.004G140500.1	651	383	651	81.72	0.56	0.41
rps7	481	338	481	Potri.011G075200.1	165	22	165	99.30	0.30	0.87
rps7	481	238	438	Potri.017G061000.1	243	30	230	90.85	0.42	0.82
rps8	405	0	343	Potri.013G140700.1	219	0	219	91.78	0.85	1.00
rps8	405	223	354	Potri.001G409700.1	183	52	183	95.42	0.32	0.72
ycf1	5502	0	975	Potri.011G073900.1	1074	0	969	98.14	0.18	0.90
ycf1	5502	3923	5064	Potri.013G075200.1	474	0	474	91.22	0.21	1.00
ycf1	5502	0	533	Potri.011G109200.1	405	0	401	83.93	0.10	0.99
ycf1	5502	666	918	Potri.T103100.1	267	0	252	100.00	0.05	0.94
ycf1	5502	4895	5137	Potri.015G131000.1	261	14	256	96.69	0.04	0.93
ycf1	5502	375	619	Potri.016G050600.1	309	0	232	93.10	0.04	0.75
ycf1	5502	3123	3339	Potri.010G052900.1	240	0	216	100.00	0.04	0.90
ycf1	5502	2472	2686	Potri.013G139700.1	204	0	204	91.67	0.04	1.00
ycf1	5502	799	1045	Potri.008G042600.1	258	4	250	86.59	0.04	0.95
ycf1	5502	162	426	Potri.010G166300.1	219	0	219	85.38	0.05	1.00
ycf1	5502	5179	5321	Potri.014G184800.1	276	120	262	97.89	0.03	0.51
ycf1	5502	1224	1388	Potri.006G084100.1	243	69	239	88.05	0.03	0.70
ycf1	5502	3087	3196	Potri.014G184800.1	276	0	110	98.17	0.02	0.40
ycf15	138	0	138	Potri.008G207400.1	138	0	138	96.38	1.00	1.00
ycf15	138	0	138	Potri.008G208800.1	138	0	138	96.38	1.00	1.00
ycf15	138	60	128	Potri.018G047600.1	180	6	74	89.71	0.49	0.38
ycf2	6859	336	6042	Potri.013G143400.1	5670	0	5655	99.77	0.83	1.00
ycf2	6859	1377	6808	Potri.013G138500.1	4689	0	4687	99.34	0.79	1.00
ycf2	6859	2136	6808	Potri.013G138500.2	3930	0	3928	99.21	0.68	1.00
ycf2	6859	5202	6859	Potri.019G028400.1	1584	0	1584	98.29	0.24	1.00
ycf2	6859	4671	5380	Potri.013G138500.3	708	0	708	99.58	0.10	1.00
ycf2	6859	1471	2115	Potri.003G067500.1	681	31	681	97.83	0.09	0.95
ycf2	6859	1990	2580	Potri.001G194800.1	612	7	594	97.79	0.09	0.96
ycf2	6859	6486	6859	Potri.011G075000.1	510	138	510	98.92	0.05	0.73
ycf2	6859	2412	3225	Potri.007G117500.1	390	0	384	89.10	0.12	0.98
ycf2	6859	6512	6859	Potri.004G128100.1	345	0	345	95.36	0.05	1.00
ycf2	6859	1797	2134	Potri.013G079300.1	348	0	337	96.14	0.05	0.97
ycf2	6859	4593	4915	Potri.004G043800.1	381	6	328	97.20	0.05	0.85
ycf2	6859	6633	6859	Potri.013G138700.1	225	0	225	98.67	0.03	1.00
ycf2	6859	5998	6242	Potri.006G162000.1	327	83	326	95.45	0.04	0.74
ycf2	6859	5998	6242	Potri.T016500.1	327	83	326	95.45	0.04	0.74
ycf2	6859	4774	5034	Potri.007G054700.1	243	5	237	84.48	0.04	0.95
ycf2	6859	2406	2565	Potri.T035700.1	210	6	165	97.48	0.02	0.76
ycf2	6859	4058	4151	Potri.007G054800.1	210	53	146	93.55	0.01	0.44
ycf3	507	0	507	Potri.003G065100.1	501	0	496	97.23	1.00	0.99
ycf3	507	126	354	Potri.013G141700.1	246	0	225	96.89	0.45	0.91
ycf3	507	0	126	Potri.013G141600.1	135	0	126	100.00	0.25	0.93
ycf4	555	0	555	Potri.T058800.1	546	0	546	97.62	1.00	1.00

Table S7 *Populus trichocarpa* NUPTs (query length 157033 bp)

Chain information			Query (cp sequence) infromation				Target (nupt) information						
**Chain score**	**Aligned length**	**Identity (%)**	**Length (bp)**	**Start**	**End**	**Gap length (bp)**	**Location**	**Chromosome length (bp)**	**Length (bp)**	**Start**	**End**	**Gap length (bp)**
10658858	121386	99.58	157033	0	2E+05	35647	Chr13	16320717	165894	1424411	1590305	44508
5436078	63241	99.37	71964	85069	2E+05	8723	Chr13	16320717	81320	1.5E+07	1.5E+07	18079
975871	10446	99.96	10490	2679	13169	44	Chr05	25890704	10452	1.5E+07	1.5E+07	6
923730	10121	98.92	13808	85341	99149	3687	Chr11	18501271	42172	7199981	7242153	32051
923730	10121	98.92	13808	1E+05	2E+05	3687	Chr11	18501271	42172	1.1E+07	1.1E+07	32051
938853	10023	99.87	10045	86026	96071	22	Chr13	16320717	10029	1.5E+07	1.5E+07	6
575997	9965	90.77	97807	34622	1E+05	87842	Chr09	12948742	751043	1E+07	1.1E+07	741078
566729	8723	94.65	115646	32826	1E+05	106923	Chr08	19465461	629440	1.5E+07	1.6E+07	620717
572196	6839	98.49	28293	17267	45560	21454	Chr03	21816808	181825	1.2E+07	1.2E+07	174986
528581	6386	94.66	6761	93690	1E+05	375	Chr08	19465461	27633	3610686	3638319	21247
513846	6143	94.92	6338	68904	75242	195	Chr17	16080358	7854	1.5E+07	1.5E+07	1711
480665	5145	99.69	5166	72739	77905	21	Chr11	18501271	5145	4637770	4642915	0
450196	4857	99.05	4865	18641	23506	8	Chr19	15942145	4858	1.3E+07	1.3E+07	1
414462	4617	99.03	55925	40668	96593	51308	Chr19	15942145	6832	3194840	3201672	2215
304157	4567	88.40	68169	21797	89966	63602	Chr03	21816808	68402	9501836	9570238	63835
413971	4396	99.95	4399	2E+05	2E+05	3	Chr13	16320717	4396	1574837	1579233	0
293718	4213	86.47	4643	97488	1E+05	430	Chr17	16080358	5568	1E+07	1E+07	1355
336052	4076	94.85	6507	1E+05	1E+05	2431	Chr04	24267051	28223	1.3E+07	1.3E+07	24147
336052	4076	94.85	6507	93995	1E+05	2431	Chr04	24267051	28223	1.1E+07	1.1E+07	24147
306665	4062	90.15	4167	70543	74710	105	Chr02	25263035	6911	1.4E+07	1.4E+07	2849
312677	3963	91.82	4109	1E+05	1E+05	146	Chr01	50495391	4082	3.5E+07	3.5E+07	119
359772	3942	98.73	3978	59814	63792	36	Chr16	14494361	3960	8215606	8219566	18
360154	3942	98.76	3978	59814	63792	36	scaffold_56	203981	3960	119309	123269	18
253397	3884	87.46	112612	37983	2E+05	108728	Chr07	15610913	6642	5719841	5726483	2758
223939	3474	84.46	32033	1E+05	1E+05	28559	Chr06	27912125	3609	6284939	6288548	135
223939	3474	84.46	32033	1E+05	1E+05	28559	Chr06	27912125	3609	2.2E+07	2.2E+07	135
52738	3117	62.82	6105	1E+05	1E+05	2988	scaffold_346	89124	4689	48175	52864	1572
52738	3117	62.82	6105	1E+05	1E+05	2988	scaffold_346	89124	4689	36260	40949	1572
219243	3078	88.69	25583	71555	97138	22505	Chr06	27912125	3392	1.1E+07	1.1E+07	314
274692	2954	99.70	2966	1E+05	2E+05	12	Chr13	16320717	2960	1582316	1585276	6
197047	2933	87.32	50698	91567	1E+05	47765	Chr07	15610913	5058	9884430	9889488	2125
197969	2867	88.59	6440	81504	87944	3573	scaffold_119	98608	19077	65515	84592	16210
243759	2814	95.63	2827	53292	56119	13	scaffold_21	931759	2816	336916	339732	2
242279	2803	95.61	2827	53292	56119	24	scaffold_21	931759	2805	435390	438195	2
191035	2740	88.36	9267	1E+05	2E+05	6527	Chr18	16958300	66592	4516562	4583154	63852
203752	2507	94.06	4912	93995	98907	2405	scaffold_23	832320	5879	336581	342460	3372
203752	2507	94.06	4912	1E+05	1E+05	2405	scaffold_23	832320	5879	489860	495739	3372
206808	2414	97.47	34738	1E+05	1E+05	32324	scaffold_81	145664	2485	70673	73158	71
38948	2396	62.81	2716	1E+05	1E+05	320	Chr14	18920894	3208	1.6E+07	1.6E+07	812
178858	2354	88.95	2419	18032	20451	65	Chr18	16958300	2391	1.2E+07	1.2E+07	37
119195	2158	79.29	2509	1E+05	1E+05	351	Chr11	18501271	2172	1.3E+07	1.3E+07	14
119195	2158	79.29	2509	96205	98714	351	Chr11	18501271	2172	5721688	5723860	14
163714	2131	89.58	2173	1E+05	1E+05	42	Chr02	25263035	2139	2.5E+07	2.5E+07	8
138522	2097	87.32	43682	36191	79873	41585	Chr16	14494361	2192	9915403	9917595	95
170025	1969	95.23	2006	40327	42333	37	Chr16	14494361	1971	7275756	7277727	2
103712	1831	80.56	2485	73330	75815	654	Chr05	25890704	1950	2.2E+07	2.2E+07	119
133698	1750	91.09	2090	1E+05	1E+05	340	scaffold_30	484195	1757	312514	314271	7
156951	1666	99.94	1667	1E+05	1E+05	1	Chr13	16320717	1666	1579380	1581046	0
118408	1623	87.92	2895	1E+05	1E+05	1272	Chr06	27912125	1631	1.7E+07	1.7E+07	8
115781	1593	87.07	1636	38283	39919	43	Chr17	16080358	1593	4587158	4588751	0
115240	1427	94.88	1478	1E+05	1E+05	51	Chr05	25890704	1970	1.2E+07	1.2E+07	543
109226	1426	90.25	1451	52295	53746	25	Chr15	15278577	1432	1.1E+07	1.1E+07	6
119204	1413	97.95	34738	96292	1E+05	33325	scaffold_81	145664	2485	72506	74991	1072
102473	1394	88.67	2715	1E+05	1E+05	1321	Chr05	25890704	3002	1.8E+07	1.8E+07	1608
82621	1392	87.00	6785	81251	88036	5393	Chr16	14494361	27805	1.3E+07	1.3E+07	26413
88997	1373	82.59	1429	2E+05	2E+05	56	Chr16	14494361	1434	1.2E+07	1.2E+07	61
94673	1356	95.72	12153	41503	53656	10797	Chr02	25263035	88252	6871960	6960212	86896
109920	1355	92.77	1382	86827	88209	27	Chr04	24267051	1360	9145304	9146664	5
109920	1355	92.77	1382	2E+05	2E+05	27	Chr04	24267051	1360	1.5E+07	1.5E+07	5
64978	1327	76.71	2619	94875	97494	1292	scaffold_99	126454	1394	93397	94791	67
64978	1327	76.71	2619	1E+05	1E+05	1292	scaffold_99	126454	1394	31663	33057	67
100593	1324	90.56	1380	75924	77304	56	Chr01	50495391	4145	3.4E+07	3.4E+07	2821
103625	1313	93.45	1585	56825	58410	272	scaffold_21	931759	1350	492222	493572	37
91787	1253	89.62	1316	66503	67819	63	Chr03	21816808	1286	1.5E+07	1.5E+07	33
110929	1240	97.58	1245	28759	30004	5	Chr12	15760346	1244	1.3E+07	1.3E+07	4
79518	1165	82.83	1226	1E+05	1E+05	61	Chr02	25263035	1171	2E+07	2E+07	6
71368	1092	89.56	21486	1E+05	1E+05	20394	Chr01	50495391	2028	4.5E+07	4.5E+07	936
38773	1049	69.11	1094	53367	54461	45	Chr10	22580532	1677	1.3E+07	1.3E+07	628
61447	1017	78.96	1047	1E+05	1E+05	30	Chr05	25890704	1021	1.3E+07	1.3E+07	4
72549	1002	88.62	1060	85130	86190	58	Chr09	12948742	1008	1.2E+07	1.2E+07	6
44313	1001	94.01	56391	22200	78591	55390	Chr18	16958300	121691	1.3E+07	1.3E+07	120690
59070	982	83.60	1242	10668	11910	260	Chr12	15760346	3698	5219456	5223154	2716
64487	969	96.70	20766	64070	84836	19797	scaffold_35	433784	69236	152642	221878	68267
80551	941	99.26	15637	1E+05	1E+05	14696	Chr10	22580532	2617	1.4E+07	1.4E+07	1676
82818	937	97.76	963	46389	47352	26	Chr10	22580532	939	8333733	8334672	2
74118	932	91.95	955	1E+05	1E+05	23	Chr19	15942145	940	1.5E+07	1.5E+07	8
58164	927	84.79	1426	12057	13483	499	Chr19	15942145	1466	1.2E+07	1.2E+07	539
17116	926	62.63	1057	1E+05	1E+05	131	scaffold_45	292166	1051	32044	33095	125
17116	926	62.63	1057	1E+05	1E+05	131	scaffold_45	292166	1051	259071	260122	125

53087	914	80.42	1990	60122	62112	1076	scaffold_995	13868	941	8303	9244	27
64908	906	90.62	978	1E+05	1E+05	72	Chr03	21816808	919	1.9E+07	1.9E+07	13
49798	873	83.05	4292	57663	61955	3419	Chr11	18501271	902	2619723	2620625	29
69965	861	92.33	872	1E+05	1E+05	11	Chr19	15942145	923	1.5E+07	1.5E+07	62
69588	829	94.33	846	34446	35292	17	Chr14	18920894	830	1934050	1934880	1
58879	815	97.42	51873	93798	1E+05	51058	scaffold_26	561009	12426	354511	366937	11611
58879	815	97.42	51873	96491	1E+05	51058	scaffold_26	561009	12426	194072	206498	11611
11622	785	63.06	2716	1E+05	1E+05	1931	scaffold_45	292166	22121	105923	128044	21336
11622	785	63.06	2716	1E+05	1E+05	1931	scaffold_45	292166	22121	164122	186243	21336
61614	781	92.96	806	3693	4499	25	Chr08	19465461	849	1.2E+07	1.2E+07	68
58432	760	90.79	760	0	760	0	Chr19	15942145	1611	1516983	1518594	851
45885	758	84.83	826	66296	67122	68	Chr01	50495391	775	1.4E+07	1.4E+07	17
40943	753	82.47	1330	60630	61960	577	Chr01	50495391	1009	1.3E+07	1.3E+07	256
42963	744	81.45	1404	1E+05	1E+05	660	Chr10	22580532	777	2.2E+07	2.2E+07	33
49365	740	85.68	886	12980	13866	146	Chr10	22580532	746	7319346	7320092	6
45012	706	86.40	744	12669	13413	38	Chr06	27912125	726	7320254	7320980	20
52636	703	93.88	19183	1E+05	1E+05	18480	Chr02	25263035	1081	2.5E+07	2.5E+07	378
46673	673	87.52	731	81346	82077	58	scaffold_119	98608	689	43365	44054	16
57923	671	95.23	672	2332	3004	1	scaffold_36	368085	671	212478	213149	0
62396	667	99.70	668	1E+05	1E+05	1	Chr13	16320717	667	1581649	1582316	0
57927	664	95.63	666	15783	16449	2	Chr09	12948742	664	2136773	2137437	0
12344	661	66.26	3798	1E+05	1E+05	3137	Chr14	18920894	21906	1.6E+07	1.6E+07	21245
52589	625	95.84	634	64070	64704	9	Chr17	16080358	626	9320985	9321611	1
40145	620	85.16	6672	1E+05	1E+05	6052	Chr11	18501271	1301	1E+07	1E+07	681
40145	620	85.16	6672	1E+05	1E+05	6052	Chr11	18501271	1301	8351365	8352666	681
44174	616	87.99	625	74017	74642	9	Chr14	18920894	836	1.2E+07	1.2E+07	220
37381	616	83.28	684	1E+05	1E+05	68	Chr10	22580532	826	1.6E+07	1.6E+07	210
38121	615	83.74	835	1E+05	1E+05	220	Chr19	15942145	619	1.3E+07	1.3E+07	4
39660	609	82.27	636	1E+05	1E+05	27	Chr15	15278577	614	1.3E+07	1.3E+07	5
43526	608	88.82	624	98313	98937	16	Chr02	25263035	614	2.1E+07	2.1E+07	6
47379	607	90.77	609	70677	71286	2	Chr02	25263035	607	1.4E+07	1.4E+07	0
56793	603	99.83	604	1E+05	1E+05	1	Chr13	16320717	603	1581046	1581649	0
41746	582	85.22	583	37678	38261	1	Chr15	15278577	582	2379026	2379608	0
42991	572	91.26	603	57671	58274	31	Chr17	16080358	583	6257820	6258403	11
45771	572	92.48	579	33586	34165	7	Chr10	22580532	581	5229622	5230203	9
46798	550	95.09	556	57854	58410	6	scaffold_21	931759	550	591485	592035	0
41710	534	91.20	536	70796	71332	2	Chr13	16320717	548	9711674	9712222	14
46117	530	97.36	532	11989	12521	2	Chr17	16080358	540	7383584	7384124	10
35925	523	84.13	528	1E+05	1E+05	5	Chr09	12948742	2033	1.3E+07	1.3E+07	1510
45374	522	97.89	531	84208	84739	9	Chr12	15760346	524	1.1E+07	1.1E+07	2
47866	522	98.66	524	96491	97015	2	Chr06	27912125	522	1.6E+07	1.6E+07	0
47866	522	98.66	524	1E+05	1E+05	2	Chr06	27912125	522	1.2E+07	1.2E+07	0
38156	515	98.06	36483	63200	99683	35968	scaffold_37	420783	1142	246706	247848	627
41094	512	92.77	514	83951	84465	2	Chr04	24267051	552	3611366	3611918	40
40921	512	93.16	536	75209	75745	24	Chr09	12948742	514	7601847	7602361	2
37433	510	91.96	540	6590	7130	30	Chr15	15278577	523	1432589	1433112	13
38861	505	90.50	541	2E+05	2E+05	36	Chr10	22580532	517	2E+07	2E+07	12
45707	500	99.40	501	1E+05	1E+05	1	Chr12	15760346	505	1.3E+07	1.3E+07	5
31422	486	83.54	499	62854	63353	13	Chr12	15760346	491	5461265	5461756	5
39915	483	92.96	483	1E+05	1E+05	0	Chr03	21816808	484	9897443	9897927	1
34166	482	87.97	490	81952	82442	8	scaffold_119	98608	485	65934	66419	3
16468	476	75.42	508	59852	60360	32	Chr14	18920894	15645	5255689	5271334	15169
38382	475	92.21	489	34819	35308	14	Chr04	24267051	475	381289	381764	0
29380	472	84.96	626	1E+05	1E+05	154	Chr18	16958300	478	1048828	1049306	6
29977	470	85.53	496	50175	50671	26	Chr01	50495391	476	9742897	9743373	6
43934	469	99.57	469	1E+05	1E+05	0	scaffold_23	832320	469	85625	86094	0
27348	467	83.94	526	90429	90955	59	scaffold_112	116414	474	21976	22450	7
27348	467	83.94	526	2E+05	2E+05	59	scaffold_112	116414	474	93964	94438	7
26827	464	85.34	535	36210	36745	71	scaffold_37	420783	465	14350	14815	1
29109	455	85.49	541	67662	68203	86	Chr16	14494361	466	8957917	8958383	11
38827	452	93.36	452	1E+05	1E+05	0	Chr19	15942145	452	1.5E+07	1.5E+07	0
34797	448	91.52	449	75461	75910	1	Chr02	25263035	450	1.2E+07	1.2E+07	2
26672	445	82.02	474	71707	72181	29	Chr01	50495391	448	1.3E+07	1.3E+07	3
26713	443	83.30	474	71707	72181	31	Chr04	24267051	457	8515297	8515754	14
25746	439	82.92	491	68916	69407	52	Chr06	27912125	829	1.1E+07	1.1E+07	390
24402	438	83.33	514	7254	7768	76	scaffold_27	548416	452	127936	128388	14
32954	434	89.40	438	20103	20541	4	Chr01	50495391	440	6813444	6813884	6
28017	431	87.94	623	69819	70442	192	scaffold_68	193879	444	99938	100382	13
26502	428	81.78	430	81016	81446	2	Chr01	50495391	432	1.4E+07	1.4E+07	4
25979	426	83.10	426	64290	64716	0	Chr02	25263035	577	8955471	8956048	151
32686	423	89.83	432	1E+05	1E+05	9	Chr10	22580532	424	1.7E+07	1.7E+07	1
22454	422	77.73	442	55733	56175	20	Chr10	22580532	431	1.4E+07	1.4E+07	9
27228	420	84.52	423	1E+05	1E+05	3	Chr08	19465461	433	5187274	5187707	13
31132	417	87.05	419	94259	94678	2	Chr05	25890704	417	7440534	7440951	0
23789	415	80.24	424	64138	64562	9	Chr12	15760346	436	9288092	9288528	21
33617	414	95.17	446	41996	42442	32	Chr15	15278577	553	3693985	3694538	139
31919	404	95.30	429	8495	8924	25	Chr15	15278577	427	3726200	3726627	23
22678	404	78.71	429	1E+05	1E+05	25	Chr19	15942145	409	1.3E+07	1.3E+07	5
11353	399	66.67	472	69782	70254	73	Chr06	27912125	405	1568337	1568742	6
26247	398	87.44	458	36281	36739	60	Chr01	50495391	398	7534358	7534756	0
27409	396	87.12	412	88058	88470	16	Chr08	19465461	396	2762232	2762628	0
27409	396	87.12	412	2E+05	2E+05	16	Chr08	19465461	396	1.7E+07	1.7E+07	0
23891	387	82.95	408	2E+05	2E+05	21	Chr03	21816808	413	1.9E+07	1.9E+07	26

32734	386	93.78	386	95292	95678	0	Chr02	25263035	387	1.4E+07	1.4E+07	1
29918	386	91.45	508	1E+05	1E+05	122	scaffold_39	362833	387	236109	236496	1
21039	382	78.80	457	77659	78116	75	Chr02	25263035	385	1.2E+07	1.2E+07	3
25385	380	83.42	381	1E+05	1E+05	1	Chr08	19465461	393	1.7E+07	1.7E+07	13
12136	380	69.47	443	76683	77126	63	Chr13	16320717	388	1.4E+07	1.4E+07	8
22148	378	83.86	487	36258	36745	109	Chr14	18920894	394	2090658	2091052	16
31350	378	95.50	380	4198	4578	2	Chr12	15760346	380	1.1E+07	1.1E+07	2
32449	378	96.03	393	1E+05	1E+05	15	Chr16	14494361	378	1.1E+07	1.1E+07	0
23109	376	80.85	422	96592	97014	46	Chr06	27912125	410	5912002	5912412	34
23109	376	80.85	422	1E+05	1E+05	46	Chr06	27912125	410	2.2E+07	2.2E+07	34
22218	376	81.91	407	1167	1574	31	Chr01	50495391	382	1623204	1623586	6
24528	376	87.77	477	36268	36745	101	Chr02	25263035	377	3989461	3989838	1
32639	373	95.71	374	73523	73897	1	Chr15	15278577	373	6329433	6329806	0
25855	372	84.41	440	1E+05	1E+05	68	Chr14	18920894	375	1.4E+07	1.4E+07	3
27237	370	88.11	373	34104	34477	3	Chr19	15942145	371	2812571	2812942	1
21502	370	78.65	397	79339	79736	27	scaffold_127	109858	370	101356	101726	0
28146	365	91.78	367	70343	70710	2	Chr01	50495391	369	2E+07	2E+07	4
27906	364	90.38	368	70257	70625	4	Chr04	24267051	367	7855819	7856186	3
25482	362	86.19	419	847	1266	57	Chr05	25890704	364	1439820	1440184	2
19984	360	81.94	439	28738	29177	79	Chr02	25263035	373	2112233	2112606	13
27436	360	91.67	370	34317	34687	10	scaffold_28	512759	366	281692	282058	6
27713	360	91.39	370	34317	34687	10	scaffold_966	14183	365	7342	7707	5
11112	360	66.67	387	1E+05	1E+05	27	Chr08	19465461	364	1.8E+07	1.8E+07	4
26647	356	91.85	378	52727	53105	22	Chr04	24267051	359	3310307	3310666	3
32042	356	99.16	1829	2E+05	2E+05	1473	Chr05	25890704	356	2.4E+07	2.4E+07	0
26751	352	92.61	370	62195	62565	18	Chr02	25263035	357	7582618	7582975	5
18784	351	80.63	362	85209	85571	11	Chr12	15760346	355	1.3E+07	1.3E+07	4
21215	349	80.23	417	1E+05	1E+05	68	Chr05	25890704	363	2.5E+07	2.5E+07	14
25547	348	89.37	357	74329	74686	9	scaffold_61	192272	348	132177	132525	0
14946	346	80.35	14914	82082	96996	14568	Chr15	15278577	366	8432226	8432592	20
27990	346	93.93	347	35964	36311	1	scaffold_1012	13660	352	9341	9693	6
21798	346	85.26	381	2E+05	2E+05	35	Chr04	24267051	350	1.8E+07	1.8E+07	4
20085	341	84.46	354	1E+05	1E+05	13	Chr01	50495391	375	4.1E+07	4.1E+07	34
22776	341	88.27	438	36281	36719	97	Chr05	25890704	359	129428	129787	18
5923	340	62.35	353	1E+05	1E+05	13	Chr10	22580532	378	1.4E+07	1.4E+07	38
26616	340	92.65	354	1E+05	1E+05	14	Chr19	15942145	343	1.3E+07	1.3E+07	3
9101	339	61.95	339	1E+05	1E+05	0	Chr01	50495391	360	4.4E+07	4.4E+07	21
25594	339	88.50	351	2E+05	2E+05	12	Chr12	15760346	339	1.5E+07	1.5E+07	0
21588	337	83.38	337	21612	21949	0	Chr02	25263035	348	1510587	1510935	11
26468	337	91.39	345	2E+05	2E+05	8	Chr07	15610913	338	5852936	5853274	1
26468	337	91.39	345	90919	91264	8	Chr07	15610913	338	9757639	9757977	1
29638	329	97.87	330	77409	77739	1	scaffold_69	214156	329	96981	97310	0
20960	327	85.02	371	2E+05	2E+05	44	scaffold_38	361808	337	347186	347523	10
20960	327	85.02	371	87637	88008	44	scaffold_38	361808	337	14285	14622	10
21818	327	86.85	348	85125	85473	21	scaffold_20	948134	328	931160	931488	1
20857	326	92.02	14635	1E+05	1E+05	14309	scaffold_126	98168	471	87332	87803	145
15016	326	76.38	345	58411	58756	19	Chr10	22580532	330	1.5E+07	1.5E+07	4
20784	326	84.36	348	85125	85473	22	scaffold_25	565605	327	16665	16992	1
27470	324	95.37	345	73728	74073	21	Chr10	22580532	324	1.1E+07	1.1E+07	0
21124	323	86.69	437	2E+05	2E+05	114	Chr04	24267051	660	2.4E+07	2.4E+07	337
21690	322	87.27	343	2E+05	2E+05	21	scaffold_20	948134	323	16646	16969	1
18027	322	77.64	340	9250	9590	18	scaffold_703	19120	322	17279	17601	0
20656	321	84.74	343	2E+05	2E+05	22	scaffold_25	565605	322	548613	548935	1
18933	320	83.44	348	50546	50894	28	Chr01	50495391	329	9682061	9682390	9
29500	319	98.75	319	36837	37156	0	scaffold_149	87786	319	26315	26634	0
19807	318	86.48	463	36281	36744	145	Chr06	27912125	319	9277675	9277994	1
20244	314	82.80	329	86810	87139	15	Chr02	25263035	316	1.5E+07	1.5E+07	2
10097	311	70.74	383	64577	64960	72	Chr14	18920894	327	3586643	3586970	16
17930	309	82.85	316	61387	61703	7	Chr14	18920894	315	2946315	2946630	6
25597	309	93.85	323	19475	19798	14	Chr07	15610913	310	9066238	9066548	1
21956	309	85.76	312	1E+05	1E+05	3	Chr10	22580532	309	1.9E+07	1.9E+07	0
26807	308	96.10	309	1E+05	1E+05	1	Chr07	15610913	308	1.4E+07	1.4E+07	0
21540	307	83.71	310	1E+05	1E+05	3	Chr01	50495391	323	3.2E+07	3.2E+07	16
16913	306	79.74	316	86487	86803	10	Chr04	24267051	1154	4748290	4749444	848
19165	302	90.40	16940	2111	19051	16638	Chr17	16080358	305	2459071	2459376	3
27340	300	97.67	300	56016	56316	0	scaffold_66	191790	300	116719	117019	0
16437	298	82.55	318	5822	6140	20	Chr15	15278577	315	271576	271891	17
21448	295	91.86	402	350	752	107	Chr12	15760346	298	37813	38111	3
27295	295	100.00	306	69807	70113	11	Chr14	18920894	295	9575469	9575764	0
18854	293	88.40	318	31737	32055	25	scaffold_24	673401	337	445077	445414	44
19694	293	86.35	317	3474	3791	24	Chr17	16080358	296	2391867	2392163	3
27558	293	99.66	293	1E+05	1E+05	0	scaffold_149	87786	293	61470	61763	0
25096	293	95.22	300	95528	95828	7	Chr01	50495391	293	4.4E+07	4.4E+07	0
23201	292	91.10	294	67846	68140	2	Chr01	50495391	292	1.3E+07	1.3E+07	0
17664	288	86.81	319	0	319	31	Chr09	12948742	315	578439	578754	27
22410	285	91.58	286	14739	15025	1	Chr06	27912125	286	8458734	8459020	1
25057	285	95.79	285	86926	87211	0	Chr14	18920894	285	6645129	6645414	0
24901	284	97.18	285	96876	97161	1	scaffold_22	1028913	289	629582	629871	5
24901	284	97.18	285	1E+05	1E+05	1	scaffold_22	1028913	289	399042	399331	5
23587	284	93.31	284	96455	96739	0	Chr02	25263035	284	2E+07	2E+07	0
24644	282	98.23	283	1E+05	1E+05	1	Chr15	15278577	1292	1.1E+07	1.1E+07	1010
20905	282	91.84	359	36281	36640	77	Chr07	15610913	282	1786779	1787061	0
26534	281	100.00	281	20011	20292	0	Chr11	18501271	281	2650811	2651092	0

21949	277	91.70	277	36038	36315	0	scaffold_609	21685	280	19626	19906	3
22471	276	92.03	276	88701	88977	0	Chr01	50495391	277	3.8E+07	3.8E+07	1
16997	275	87.64	317	28772	29089	42	scaffold_1162	11758	279	10285	10564	4
24840	275	97.45	275	1E+05	1E+05	0	scaffold_37	420783	275	172935	173210	0
21843	273	91.21	273	36038	36311	0	Chr15	15278577	273	7665326	7665599	0
21626	271	92.62	271	1E+05	1E+05	0	Chr15	15278577	272	1.4E+07	1.4E+07	1
22874	271	94.46	275	13486	13761	4	scaffold_172	83343	271	72404	72675	0
19905	270	87.78	302	1E+05	1E+05	32	Chr07	15610913	288	1.4E+07	1.4E+07	18
24878	270	98.15	270	16118	16388	0	scaffold_82	146869	270	129749	130019	0
13140	269	78.81	280	4235	4515	11	Chr16	14494361	361	344920	345281	92
20024	268	91.79	288	1E+05	1E+05	20	Chr15	15278577	284	1.4E+07	1.4E+07	16
21097	264	89.77	264	1E+05	1E+05	0	Chr02	25263035	265	1.8E+07	1.8E+07	1
14395	261	78.16	287	2E+05	2E+05	26	Chr17	16080358	261	1.6E+07	1.6E+07	0
21989	259	95.37	264	75665	75929	5	scaffold_22	1028913	259	396425	396684	0
22308	259	96.14	259	1E+05	1E+05	0	Chr14	18920894	259	1.6E+07	1.6E+07	0
18914	258	90.31	341	1E+05	1E+05	83	scaffold_2073	4915	258	3374	3632	0
18914	258	90.31	341	92534	92875	83	scaffold_2073	4915	258	1283	1541	0
14831	254	85.83	304	2E+05	2E+05	50	Chr07	15610913	266	1.4E+07	1.4E+07	12
8875	252	66.27	268	80775	81043	16	Chr06	27912125	259	2905790	2906049	7
13478	250	81.20	253	96496	96749	3	Chr17	16080358	272	1.5E+07	1.5E+07	22
21163	248	96.77	248	90131	90379	0	scaffold_36	368085	1783	284104	285887	1535
17078	248	89.11	326	2E+05	2E+05	78	Chr05	25890704	248	2.6E+07	2.6E+07	0
13633	247	78.54	248	19422	19670	1	Chr10	22580532	255	8454080	8454335	8
11141	247	76.52	294	98878	99172	47	scaffold_916	15225	251	9426	9677	4
11141	247	76.52	294	1E+05	1E+05	47	scaffold_916	15225	251	5548	5799	4
13612	239	79.92	259	11208	11467	20	Chr06	27912125	247	3286170	3286417	8
14299	239	81.17	249	1E+05	1E+05	10	scaffold_158	78621	243	8121	8364	4
14299	239	81.17	249	1E+05	1E+05	10	scaffold_158	78621	243	70257	70500	4
16988	238	86.13	246	1E+05	1E+05	8	Chr05	25890704	238	2.4E+07	2.4E+07	0
22073	238	98.74	238	1E+05	1E+05	0	Chr08	19465461	238	3758550	3758788	0
13315	237	78.06	265	2E+05	2E+05	28	scaffold_146	91718	237	10808	11045	0
13315	237	78.06	265	87587	87852	28	scaffold_146	91718	237	80673	80910	0
9651	236	73.73	265	57037	57302	29	Chr10	22580532	251	9324335	9324586	15
12097	236	76.69	300	2E+05	2E+05	64	Chr19	15942145	241	1.6E+07	1.6E+07	5
19272	236	93.64	257	1E+05	1E+05	21	scaffold_172	83343	237	10934	11171	1
19255	236	93.64	240	16377	16617	4	scaffold_156	82369	236	32142	32378	0
16009	234	100.00	24685	66818	91503	24451	Chr05	25890704	234	6288151	6288385	0
7562	233	69.96	258	94918	95176	25	Chr14	18920894	255	7172562	7172817	22
18847	231	95.24	238	17073	17311	7	Chr11	18501271	234	975262	975496	3
11008	231	74.03	242	1E+05	1E+05	11	Chr17	16080358	231	1.5E+07	1.5E+07	0
16233	230	99.13	19928	56534	76462	19698	scaffold_22	1028913	230	38130	38360	0
17537	229	91.70	230	1E+05	1E+05	1	Chr01	50495391	235	2.8E+07	2.8E+07	6
14604	229	85.15	237	73155	73392	8	scaffold_467	30920	233	572	805	4
8114	226	64.60	229	80098	80327	3	Chr06	27912125	230	2776755	2776985	4
17733	226	89.82	227	68609	68836	1	scaffold_207	68427	226	31828	32054	0
7842	224	75.89	270	1E+05	1E+05	46	scaffold_1213	11155	230	2058	2288	6
18699	224	96.43	225	27661	27886	1	Chr05	25890704	229	2E+07	2E+07	5
13281	223	86.10	248	5758	6006	25	scaffold_1153	13338	224	305	529	1
14841	221	85.07	232	99874	1E+05	11	scaffold_73	173458	222	83659	83881	1
14841	221	85.07	232	1E+05	1E+05	11	scaffold_73	173458	222	89577	89799	1
17970	220	93.64	247	1E+05	1E+05	27	Chr16	14494361	220	1.3E+07	1.3E+07	0
16101	218	86.24	218	85330	85548	0	Chr01	50495391	219	3.7E+07	3.7E+07	1
12410	217	85.25	224	44862	45086	7	scaffold_104	146719	224	52046	52270	7
13167	216	81.48	226	2E+05	2E+05	10	scaffold_61	192272	217	189295	189512	1
15204	212	85.85	212	87588	87800	0	Chr16	14494361	214	1.3E+07	1.3E+07	2
10084	212	74.06	223	1E+05	1E+05	11	scaffold_86	151614	212	40993	41205	0
15833	212	88.68	218	77224	77442	6	Chr05	25890704	212	2.3E+07	2.3E+07	0
10084	212	74.06	223	1E+05	1E+05	11	scaffold_86	151614	212	110409	110621	0
10378	211	77.25	219	33377	33596	8	Chr16	14494361	222	4024610	4024832	11
9963	206	77.18	231	2E+05	2E+05	25	Chr04	24267051	211	2.2E+07	2.2E+07	5
16522	205	94.15	218	18257	18475	13	Chr04	24267051	205	1675672	1675877	0
16006	203	92.12	203	1E+05	1E+05	0	Chr08	19465461	226	1.9E+07	1.9E+07	23
12897	201	82.59	211	22831	23042	10	Chr02	25263035	209	1882582	1882791	8
12273	201	85.57	212	1E+05	1E+05	11	Chr10	22580532	206	1.5E+07	1.5E+07	5
12796	200	87.50	234	13516	13750	34	Chr07	15610913	203	6911193	6911396	3
12735	200	80.50	202	86548	86750	2	Chr17	16080358	201	1E+07	1E+07	1
13898	199	88.94	205	16385	16590	6	Chr03	21816808	203	5058628	5058831	4
14083	199	85.43	301	1E+05	1E+05	102	Chr14	18920894	199	1.4E+07	1.4E+07	0
7622	198	75.25	200	69001	69201	2	Chr09	12948742	225	7148456	7148681	27
8099	198	78.28	230	27645	27875	32	scaffold_954	14280	203	782	985	5
13901	198	89.39	206	1E+05	2E+05	8	Chr03	21816808	199	1.4E+07	1.4E+07	1
17735	198	97.47	198	17638	17836	0	Chr10	22580532	198	5023351	5023549	0
18104	195	98.97	195	1E+05	1E+05	0	Chr19	15942145	195	1.4E+07	1.4E+07	0
10920	193	82.90	221	98509	98730	28	Chr14	18920894	198	1.3E+07	1.3E+07	5
13020	191	85.86	195	67554	67749	4	Chr10	22580532	191	1.6E+07	1.6E+07	0
12886	191	84.82	205	2E+05	2E+05	14	Chr06	27912125	191	2.3E+07	2.3E+07	0
8074	190	73.16	212	60706	60918	22	Chr13	16320717	198	6367108	6367306	8
10907	190	84.21	225	51162	51387	35	Chr11	18501271	190	4047865	4048055	0
12072	189	82.54	190	1E+05	1E+05	1	scaffold_1534	8078	192	3116	3308	3
12012	188	86.70	198	41828	42026	10	scaffold_576	23531	231	4318	4549	43
11352	186	86.02	225	2E+05	2E+05	39	Chr14	18920894	186	1.9E+07	1.9E+07	0
12459	185	82.70	185	1E+05	1E+05	0	Chr19	15942145	189	1.3E+07	1.3E+07	4
9723	185	77.30	193	35279	35472	8	Chr05	25890704	186	2E+07	2E+07	1

15827	185	97.84	188	1E+05	1E+05	3	scaffold_28	512759	185	127724	127909	0
15661	184	95.65	186	1108	1294	2	Chr18	16958300	184	7628133	7628317	0
14573	183	92.90	195	17066	17261	12	Chr15	15278577	183	5061544	5061727	0
10497	179	83.24	180	98404	98584	1	scaffold_528	25539	190	6065	6255	11
10497	179	83.24	180	1E+05	1E+05	1	scaffold_528	25539	190	19284	19474	11
14078	179	90.50	179	1E+05	1E+05	0	scaffold_126	98168	187	10365	10552	8
7819	179	81.56	15452	1E+05	1E+05	15273	scaffold_27	548416	179	520677	520856	0
11053	176	85.80	191	57455	57646	15	scaffold_479	29137	177	6577	6754	1
8998	176	89.77	16782	1E+05	1E+05	16606	Chr14	18920894	177	1.5E+07	1.5E+07	1
14973	176	94.89	176	29214	29390	0	Chr16	14494361	176	2448480	2448656	0
11391	175	87.43	179	75231	75410	4	Chr07	15610913	182	9107717	9107899	7
10555	175	79.43	176	39372	39548	1	Chr16	14494361	175	5854704	5854879	0
7784	174	78.16	180	27694	27874	6	Chr01	50495391	198	7159531	7159729	24
10204	171	83.63	173	6466	6639	2	Chr10	22580532	179	5429217	5429396	8
9285	170	81.76	178	17479	17657	8	Chr03	21816808	181	8200411	8200592	11
14531	170	93.53	170	1E+05	1E+05	0	Chr18	16958300	170	3798300	3798470	0
12715	170	92.35	176	1497	1673	6	scaffold_141	115850	170	33707	33877	0
8231	169	79.29	203	9473	9676	34	Chr16	14494361	170	653986	654156	1
11085	167	88.62	175	97756	97931	8	Chr14	18920894	174	1.2E+07	1.2E+07	7
15095	167	96.41	167	1E+05	1E+05	0	Chr06	27912125	167	2.1E+07	2.1E+07	0
15095	167	96.41	167	1E+05	1E+05	0	Chr06	27912125	167	6690619	6690786	0
13479	166	93.98	166	54882	55048	0	Chr09	12948742	167	3384364	3384531	1
13366	165	95.76	178	27305	27483	13	Chr01	50495391	165	3218464	3218629	0
11964	164	85.98	164	91622	91786	0	Chr16	14494361	164	1E+07	1E+07	0
6678	162	75.93	165	24672	24837	3	Chr08	19465461	184	1.3E+07	1.3E+07	22
7909	162	75.31	195	2E+05	2E+05	33	Chr03	21816808	162	1.6E+07	1.6E+07	0
9656	159	80.50	160	76804	76964	1	Chr06	27912125	198	1885833	1886031	39
11367	157	89.17	166	86417	86583	9	Chr06	27912125	157	3596853	3597010	0
11367	157	89.17	166	2E+05	2E+05	9	Chr06	27912125	157	2.4E+07	2.4E+07	0
7306	155	76.13	169	78785	78954	14	Chr05	25890704	157	2.4E+07	2.4E+07	2
11213	155	88.39	157	73202	73359	2	Chr11	18501271	155	5513446	5513601	0
7658	150	76.67	162	60716	60878	12	scaffold_256	53559	154	40341	40495	4
7467	149	77.85	173	68348	68521	24	Chr10	22580532	160	1.6E+07	1.6E+07	11
11221	149	86.58	149	93909	94058	0	Chr15	15278577	149	1.2E+07	1.2E+07	0
10885	147	87.07	147	1E+05	1E+05	0	Chr04	24267051	147	1.3E+07	1.3E+07	0
7921	145	76.55	145	72728	72873	0	scaffold_1644	7488	235	6765	7000	90
7318	145	75.86	146	34070	34216	1	Chr16	14494361	151	8637926	8638077	6
7570	145	78.62	145	57545	57690	0	Chr13	16320717	150	1871927	1872077	5
9162	145	86.21	150	1E+05	1E+05	5	Chr12	15760346	147	1.3E+07	1.3E+07	2
11949	144	94.44	144	2E+05	2E+05	0	Chr04	24267051	150	2.3E+07	2.3E+07	6
11220	144	90.97	144	1E+05	1E+05	0	Chr05	25890704	149	2.5E+07	2.5E+07	5
10896	144	87.50	144	1E+05	1E+05	0	Chr18	16958300	144	1.6E+07	1.6E+07	0
7524	143	76.92	158	1E+05	1E+05	15	Chr04	24267051	149	1.3E+07	1.3E+07	6
7524	143	76.92	158	1E+05	1E+05	15	Chr04	24267051	149	1.1E+07	1.1E+07	6
7653	143	76.92	144	41183	41327	1	scaffold_608	21707	146	19565	19711	3
9662	143	82.52	177	85851	86028	34	Chr16	14494361	143	9980235	9980378	0
10073	141	91.49	150	1E+05	1E+05	9	scaffold_185	88296	154	52172	52326	13
10073	141	91.49	150	1E+05	1E+05	9	scaffold_185	88296	154	35970	36124	13
9013	140	82.14	140	1E+05	1E+05	0	scaffold_112	116414	140	106445	106585	0
8957	139	86.33	143	65607	65750	4	scaffold_31	440175	140	18585	18725	1
6146	138	77.54	154	61844	61998	16	scaffold_270	51148	139	18076	18215	1
11599	138	94.20	139	10595	10734	1	Chr03	21816808	138	1386947	1387085	0
8380	138	83.33	147	1E+05	1E+05	9	scaffold_397	35893	138	34594	34732	0
8380	138	83.33	147	1E+05	1E+05	9	scaffold_397	35893	138	1161	1299	0
9183	134	87.31	134	1E+05	1E+05	0	scaffold_1378	9943	152	8738	8890	18
7309	134	73.13	134	1E+05	1E+05	0	Chr04	24267051	134	1.5E+07	1.5E+07	0
7309	134	73.13	134	96452	96586	0	Chr04	24267051	134	9595129	9595263	0
10509	134	89.55	137	37218	37355	3	Chr18	16958300	134	653145	653279	0
6903	134	80.60	161	2E+05	2E+05	27	Chr01	50495391	134	5E+07	5E+07	0
6679	133	84.96	4433	1E+05	1E+05	4300	scaffold_222	67768	1434	20462	21896	1301
6168	132	76.52	134	75431	75565	2	Chr14	18920894	145	6299673	6299818	13
6298	130	75.38	134	1E+05	1E+05	4	scaffold_576	23531	132	18994	19126	2
8360	129	83.72	134	10689	10823	5	Chr19	15942145	129	7131821	7131950	0
7649	128	81.25	131	1E+05	1E+05	3	Chr12	15760346	130	1.3E+07	1.3E+07	2
7529	127	83.46	163	1E+05	1E+05	36	Chr19	15942145	127	1.2E+07	1.2E+07	0
6393	126	79.37	129	8402	8531	3	scaffold_1228	10985	141	10684	10825	15
4855	126	72.22	127	22387	22514	1	scaffold_92	132020	130	71733	71863	4
5644	126	72.22	126	1E+05	1E+05	0	Chr18	16958300	129	1.4E+07	1.4E+07	3
8213	126	80.16	126	1E+05	1E+05	0	Chr02	25263035	127	2.5E+07	2.5E+07	1
9753	125	90.40	125	85703	85828	0	Chr17	16080358	129	1E+07	1E+07	4
7354	122	78.69	127	1E+05	1E+05	5	scaffold_127	109858	123	105941	106064	1
7354	122	78.69	127	1E+05	1E+05	5	scaffold_127	109858	123	3794	3917	1
9156	121	90.91	121	1E+05	1E+05	0	scaffold_946	14435	145	9821	9966	24
9275	120	87.50	120	33376	33496	0	scaffold_99	126454	120	12874	12994	0
9257	120	89.17	120	33376	33496	0	scaffold_449	31428	120	28901	29021	0
10218	120	92.50	120	33376	33496	0	Chr11	18501271	120	2065446	2065566	0
10397	120	94.17	120	33376	33496	0	Chr06	27912125	120	9231367	9231487	0
9273	120	87.50	120	33376	33496	0	scaffold_131	95303	120	15727	15847	0
9703	120	89.17	120	33376	33496	0	scaffold_2185	4801	120	3586	3706	0
7334	120	81.67	132	32603	32735	12	scaffold_1446	8983	120	642	762	0
9067	120	85.83	120	1E+05	1E+05	0	Chr02	25263035	120	2.3E+07	2.3E+07	0
9746	119	98.32	119	1E+05	1E+05	0	Chr19	15942145	133	1.3E+07	1.3E+07	14
9615	119	97.48	119	1E+05	1E+05	0	scaffold_50	254906	133	156877	157010	14

10560	119	98.32	119	1E+05	1E+05	0	scaffold_50	254906	128	135788	135916	9
10560	119	98.32	119	1E+05	1E+05	0	scaffold_50	254906	128	118990	119118	9
7218	119	82.35	119	27755	27874	0	scaffold_41	316090	119	238656	238775	0
8468	119	84.03	120	1E+05	1E+05	1	Chr03	21816808	119	1.6E+07	1.6E+07	0
9830	119	91.60	119	33377	33496	0	scaffold_135	94190	119	78382	78501	0
9828	119	91.60	119	33376	33495	0	scaffold_34	446712	119	240843	240962	0
8073	118	85.59	118	85793	85911	0	scaffold_61	192272	121	187656	187777	3
9604	118	90.68	118	63178	63296	0	Chr14	18920894	118	8615830	8615948	0
10294	118	94.92	118	57731	57849	0	scaffold_21	931759	118	591367	591485	0
8248	118	81.36	118	1E+05	1E+05	0	scaffold_1446	8983	118	8336	8454	0
2663	116	69.83	129	1E+05	1E+05	13	Chr09	12948742	132	1.1E+07	1.1E+07	16
6641	116	81.03	117	1E+05	1E+05	1	scaffold_2268	4409	122	2923	3045	6
6641	116	81.03	117	1E+05	1E+05	1	scaffold_2268	4409	122	1364	1486	6
8304	116	90.52	126	2E+05	2E+05	10	scaffold_28	512759	118	181249	181367	2
6607	116	79.31	119	1E+05	1E+05	3	scaffold_50	254906	118	240927	241045	2
8304	116	90.52	126	87748	87874	10	scaffold_28	512759	118	331392	331510	2
6607	116	79.31	119	1E+05	1E+05	3	scaffold_50	254906	118	13861	13979	2
8072	116	82.76	116	33378	33494	0	scaffold_150	90931	116	21488	21604	0
6772	114	85.96	137	2E+05	2E+05	23	Chr11	18501271	117	1.7E+07	1.7E+07	3
6805	114	76.32	114	85677	85791	0	Chr01	50495391	114	1.5E+07	1.5E+07	0
4988	114	72.81	121	2E+05	2E+05	7	Chr03	21816808	114	1.6E+07	1.6E+07	0
5968	113	84.07	134	43003	43137	21	Chr03	21816808	128	1.2E+07	1.2E+07	15
6824	113	86.73	143	98024	98167	30	Chr19	15942145	123	8173443	8173566	10
10782	113	99.12	113	1E+05	1E+05	0	Chr03	21816808	113	1.8E+07	1.8E+07	0
9453	112	95.54	113	60111	60224	1	scaffold_1446	8983	112	7304	7416	0
5597	112	75.89	115	1E+05	1E+05	3	Chr12	15760346	112	1.4E+07	1.4E+07	0
6144	111	86.49	113	82242	82355	2	scaffold_42	309264	138	204365	204503	27
9198	110	91.82	110	1E+05	1E+05	0	Chr08	19465461	110	7518772	7518882	0
3988	109	70.64	113	44023	44136	4	Chr13	16320717	113	110109	110222	4
6518	109	81.65	109	1E+05	1E+05	0	Chr02	25263035	111	2.5E+07	2.5E+07	2
7727	108	83.33	108	97275	97383	0	scaffold_1228	10985	108	10756	10864	0
7727	108	83.33	108	1E+05	1E+05	0	scaffold_1228	10985	108	121	229	0
6990	107	83.18	107	69567	69674	0	Chr02	25263035	108	1E+07	1E+07	1
6722	106	82.08	106	37166	37272	0	Chr15	15278577	107	1531203	1531310	1
7423	104	88.46	104	22867	22971	0	scaffold_1935	5379	106	4216	4322	2
5502	104	75.96	113	64317	64430	9	Chr02	25263035	104	9218962	9219066	0
5556	101	85.15	106	2E+05	2E+05	5	Chr18	16958300	105	1.6E+07	1.6E+07	4
6566	101	80.20	101	1E+05	1E+05	0	Chr16	14494361	101	1.1E+07	1.1E+07	0
2850	99	71.72	112	36314	36426	13	Chr09	12948742	99	1E+07	1E+07	0
4679	97	74.23	101	1E+05	1E+05	4	scaffold_128	99216	98	82482	82580	1
4679	97	74.23	101	1E+05	1E+05	4	scaffold_128	99216	98	16636	16734	1
5293	97	80.41	101	48482	48583	4	Chr14	18920894	97	4885352	4885449	0
7105	95	89.47	95	45910	46005	0	Chr10	22580532	95	6910470	6910565	0
5395	95	78.95	102	87284	87386	7	Chr02	25263035	95	1.8E+07	1.8E+07	0
4307	95	69.47	95	75781	75876	0	Chr01	50495391	95	3.1E+07	3.1E+07	0
6199	94	84.04	94	32829	32923	0	Chr01	50495391	101	3456074	3456175	7
8180	94	97.87	100	1359	1459	6	Chr10	22580532	94	715767	715861	0
5416	94	74.47	94	49677	49771	0	scaffold_123	95485	94	45909	46003	0
6310	93	79.57	93	1E+05	1E+05	0	Chr10	22580532	93	1.2E+07	1.2E+07	0
7647	93	91.40	93	50391	50484	0	scaffold_223	63517	93	21763	21856	0
6441	93	83.87	93	1E+05	1E+05	0	Chr01	50495391	93	4.9E+07	4.9E+07	0
7097	93	90.32	94	46497	46591	1	Chr05	25890704	93	230375	230468	0
8116	92	95.65	92	84076	84168	0	scaffold_233	60227	92	56475	56567	0
6813	92	88.04	92	24709	24801	0	Chr16	14494361	92	3517887	3517979	0
6402	91	90.11	120	48927	49047	29	Chr09	12948742	91	3237654	3237745	0
5919	91	85.71	97	69273	69370	6	Chr01	50495391	91	1.6E+07	1.6E+07	0
8101	90	96.67	90	1E+05	1E+05	0	Chr05	25890704	90	1.2E+07	1.2E+07	0
5197	86	86.05	91	59243	59334	5	scaffold_222	67768	87	45936	46023	1
4575	85	80.00	87	38148	38235	2	Chr01	50495391	86	9616380	9616466	1
4941	85	76.47	85	78230	78315	0	scaffold_29	527496	85	493233	493318	0
5335	84	82.14	84	1E+05	1E+05	0	scaffold_250	55372	88	51531	51619	4
5335	84	82.14	84	1E+05	1E+05	0	scaffold_250	55372	88	3753	3841	4
4525	84	79.76	90	31686	31776	6	scaffold_39	362833	85	144538	144623	1
7307	84	95.24	84	70568	70652	0	Chr02	25263035	84	1.4E+07	1.4E+07	0
5448	84	83.33	85	78230	78315	1	scaffold_20	948134	84	646959	647043	0
5824	83	90.36	84	50587	50671	1	Chr01	50495391	114	9743504	9743618	31
5830	81	90.12	81	92661	92742	0	Chr12	15760346	120	1.3E+07	1.3E+07	39
6940	81	95.06	81	1E+05	1E+05	0	Chr14	18920894	81	1.7E+07	1.7E+07	0
1678	81	66.67	89	36225	36314	8	Chr09	12948742	81	1E+07	1E+07	0
5655	81	88.89	101	1E+05	1E+05	20	scaffold_86	151614	81	133196	133277	0
5655	81	88.89	101	1E+05	1E+05	20	scaffold_86	151614	81	18337	18418	0
5655	81	88.89	101	1E+05	1E+05	20	Chr17	16080358	81	1.5E+07	1.5E+07	0
6032	80	92.50	81	93101	93182	1	Chr04	24267051	82	9926410	9926492	2
6032	80	92.50	81	1E+05	1E+05	1	Chr04	24267051	82	1.4E+07	1.4E+07	2
4410	80	75.00	80	95291	95371	0	scaffold_1470	8685	80	2437	2517	0
5486	80	83.75	80	23477	23557	0	Chr09	12948742	80	9711087	9711167	0
6347	80	93.75	107	2E+05	2E+05	27	scaffold_1402	9486	80	1142	1222	0
6435	80	90.00	80	95674	95754	0	scaffold_93	128341	80	89328	89408	0
4410	80	75.00	80	1E+05	1E+05	0	scaffold_1470	8685	80	6168	6248	0
6435	80	90.00	80	1E+05	1E+05	0	scaffold_93	128341	80	38933	39013	0
6347	80	93.75	107	89372	89479	27	scaffold_1402	9486	80	8264	8344	0
6390	79	94.94	83	2E+05	2E+05	4	Chr11	18501271	79	1.1E+07	1.1E+07	0
6390	79	94.94	83	87203	87286	4	Chr11	18501271	79	7207580	7207659	0

5757	79	84.81	79	1137	1216	0	Chr05	25890704	79	1467937	1468016	0
1370	78	62.82	84	1E+05	1E+05	6	Chr06	27912125	82	2.2E+07	2.2E+07	4
6195	78	91.03	78	1E+05	1E+05	0	Chr02	25263035	81	2.4E+07	2.4E+07	3
4457	78	79.49	92	1E+05	1E+05	14	Chr15	15278577	78	1.3E+07	1.3E+07	0
4958	78	79.49	78	50527	50605	0	Chr13	16320717	78	865391	865469	0
4921	76	84.21	76	1017	1093	0	Chr05	25890704	80	1467857	1467937	4
4505	75	84.00	75	72360	72435	0	Chr01	50495391	80	2.2E+07	2.2E+07	5
4130	74	78.38	74	16960	17034	0	scaffold_42	309264	75	97974	98049	1
5737	74	90.54	74	99579	99653	0	Chr06	27912125	74	1.7E+07	1.7E+07	0
5737	74	90.54	74	1E+05	1E+05	0	Chr06	27912125	74	1.1E+07	1.1E+07	0
4304	73	86.30	73	71362	71435	0	Chr02	25263035	106	1.4E+07	1.4E+07	33
4388	73	80.82	73	17881	17954	0	Chr11	18501271	77	497990	498067	4
4730	73	82.19	73	18303	18376	0	Chr09	12948742	73	9261784	9261857	0
4900	73	84.93	73	55117	55190	0	Chr01	50495391	73	1.2E+07	1.2E+07	0
3687	73	71.23	73	69898	69971	0	Chr02	25263035	73	1.1E+07	1.1E+07	0
3790	71	74.65	71	2E+05	2E+05	0	Chr03	21816808	71	1.9E+07	1.9E+07	0
4996	70	85.71	70	358	428	0	Chr03	21816808	70	381860	381930	0
5736	70	92.86	70	87874	87944	0	Chr16	14494361	70	1.3E+07	1.3E+07	0
5132	69	86.96	69	1E+05	1E+05	0	Chr11	18501271	69	7142633	7142702	0
3587	67	76.12	67	1E+05	1E+05	0	scaffold_222	67768	71	47235	47306	4
4015	67	79.10	67	99697	99764	0	Chr05	25890704	68	2.5E+07	2.5E+07	1
5706	67	94.03	67	33417	33484	0	scaffold_2070	4937	67	632	699	0
4246	67	79.10	67	2E+05	2E+05	0	Chr06	27912125	67	1.8E+07	1.8E+07	0
5266	67	89.55	67	682	749	0	Chr19	15942145	67	2568378	2568445	0
5006	67	91.04	70	39550	39620	3	Chr02	25263035	67	4702482	4702549	0
5814	67	94.03	67	1E+05	1E+05	0	Chr03	21816808	67	1.9E+07	1.9E+07	0
4449	66	86.36	66	1E+05	1E+05	0	scaffold_53	257254	75	257042	257117	9
4006	66	86.36	74	64637	64711	8	Chr13	16320717	66	1489167	1489233	0
1318	66	63.64	80	69046	69126	14	Chr06	27912125	66	1.1E+07	1.1E+07	0
4959	66	87.88	66	1E+05	1E+05	0	Chr02	25263035	66	1.9E+07	1.9E+07	0
1194	65	63.08	66	12905	12971	1	Chr19	15942145	72	1.2E+07	1.2E+07	7
5278	65	90.77	65	22906	22971	0	Chr16	14494361	65	1309745	1309810	0
3628	65	76.92	65	42516	42581	0	Chr03	21816808	65	1.2E+07	1.2E+07	0
5275	65	89.23	65	1E+05	1E+05	0	Chr10	22580532	65	1.1E+07	1.1E+07	0
3973	64	78.13	64	1E+05	1E+05	0	Chr10	22580532	64	1.3E+07	1.3E+07	0
4595	63	88.89	63	16948	17011	0	scaffold_25	565605	64	159917	159981	1
3785	62	82.26	62	28986	29048	0	scaffold_250	55372	62	17072	17134	0
3999	62	83.87	62	28986	29048	0	Chr18	16958300	62	325939	326001	0
1123	61	62.30	62	68978	69040	1	Chr06	27912125	69	1.1E+07	1.1E+07	8
5366	61	96.72	61	34868	34929	0	scaffold_53	257254	61	190433	190494	0
4973	61	91.80	61	25238	25299	0	Chr04	24267051	61	142514	142575	0
1913	60	71.67	65	42581	42646	5	Chr03	21816808	60	1.2E+07	1.2E+07	0
3807	60	81.67	60	4104	4164	0	Chr06	27912125	60	501020	501080	0
4313	59	86.44	59	1E+05	1E+05	0	scaffold_576	23531	59	6167	6226	0
4705	59	96.61	69	1E+05	1E+05	10	Chr12	15760346	59	1.6E+07	1.6E+07	0
4313	59	86.44	59	93715	93774	0	scaffold_576	23531	59	17305	17364	0
1428	59	61.02	59	1E+05	1E+05	0	Chr06	27912125	59	6286635	6286694	0
3585	58	81.03	58	1E+05	1E+05	0	scaffold_156	82369	58	81213	81271	0
3650	58	77.59	58	1E+05	1E+05	0	Chr15	15278577	58	1.1E+07	1.1E+07	0
3710	55	85.45	55	2114	2169	0	Chr10	22580532	57	4453370	4453427	2
3220	55	85.45	60	71302	71362	5	Chr02	25263035	55	1.4E+07	1.4E+07	0
1145	55	72.73	60	36415	36475	5	Chr05	25890704	55	129506	129561	0
4668	55	92.73	55	39446	39501	0	Chr17	16080358	55	8777827	8777882	0
3618	54	81.48	54	1E+05	1E+05	0	Chr05	25890704	54	2.6E+07	2.6E+07	0
3937	54	87.04	54	25324	25378	0	Chr16	14494361	54	1426949	1427003	0
2385	52	78.85	52	36346	36398	0	Chr05	25890704	56	129450	129506	4
1958	52	75.00	69	64699	64768	17	Chr13	16320717	52	1489287	1489339	0
4920	51	100.00	51	1E+05	1E+05	0	scaffold_63	189656	51	36529	36580	0
4920	51	100.00	51	1E+05	1E+05	0	scaffold_63	189656	51	153076	153127	0
1992	50	78.00	54	36426	36480	4	Chr09	12948742	50	1E+07	1E+07	0
3633	50	90.00	50	35200	35250	0	Chr01	50495391	50	8356455	8356505	0
3784	48	91.67	48	1E+05	1E+05	0	scaffold_116	103364	48	91211	91259	0
3674	47	89.36	47	18408	18455	0	Chr15	15278577	47	6846414	6846461	0
3203	46	86.96	46	42647	42693	0	Chr03	21816808	46	1.2E+07	1.2E+07	0
4339	46	100.00	46	90959	91005	0	Chr01	50495391	46	1.6E+07	1.6E+07	0
3430	46	86.96	46	1E+05	1E+05	0	Chr04	24267051	46	1.2E+07	1.2E+07	0
1811	45	80.00	55	36498	36553	10	Chr09	12948742	45	1E+07	1E+07	0
3434	45	88.89	45	1E+05	1E+05	0	Chr14	18920894	45	1.7E+07	1.7E+07	0
1444	44	77.27	47	36578	36625	3	Chr05	25890704	44	129629	129673	0
3585	42	95.24	42	90635	90677	0	Chr02	25263035	42	1.9E+07	1.9E+07	0
1757	42	80.95	48	36584	36632	6	Chr09	12948742	42	1E+07	1E+07	0
3502	41	100.00	47	64844	64891	6	Chr13	16320717	41	1489408	1489449	0
1472	37	78.38	78	36480	36558	41	Chr05	25890704	37	129592	129629	0
3008	37	91.89	37	1229	1266	0	Chr05	25890704	37	1468016	1468053	0
2744	37	97.30	43	64584	64627	6	Chr13	16320717	37	1489159	1489196	0
3289	36	97.22	36	1E+05	1E+05	0	Chr02	25263035	36	1.6E+07	1.6E+07	0
1918	33	78.79	33	50549	50582	0	Chr01	50495391	33	9743471	9743504	0
1961	32	90.63	45	43165	43210	13	Chr03	21816808	32	1.2E+07	1.2E+07	0
1851	27	88.89	27	64770	64797	0	Chr13	16320717	27	1489339	1489366	0
2076	24	95.83	24	43275	43299	0	Chr03	21816808	24	1.2E+07	1.2E+07	0

Note: Number of NUPT in Populus trichocarpa with length and identity no less than 100 bp and 90% , respectively, as is the criteria of Yoshida et al. (2013), is 175, and correspoding total length is 388664bp (all NUPTs length is 536306bp).

Table S8 List of multiple cp genes hitted to same nucleus gene in *Populus trichocarpa*

Nucleus gene ID	Order of nupts in nucleus	Gene hitted in CDS	In order of cp genome?	Location
Potri.002G237300	*psbE-pebF-psbL*	*psbE & psbL*	No	Chr02:23019316-23019874
Potri.003G067400	*psaJ-rpl33*	*rpl33*	Yes	Chr03:9567815-9569026
Potri.005G154300	*trnI-rpl23-rpl2*	*rpl2*	Yes	Chr05:14813787-14815682
Potri.005G154500	*trnQ* ^{UUG} *-psbK-psbI-trnS* ^{GCU}	*psbK*	Yes	Chr05:14818203-14820092
Potri.009G170600	*trnE* ^{UUC} *-trnY*	**none**	Yes	Chr09:12917243-12934717
Potri.010G052900	*ndhA-ndhH-rps15-ycf1*	*ycf1*	No	Chr10:8333019-8333763
Potri.011G074200	*trnA* ^{UGC} *-rps19*	*rps19*	No	Chr11:7199187-7200267
Potri.011G075000	*16S_rRNA-trnI* ^{GAU}	*ycf2*	Yes	Chr11:7215822-7217613
Potri.011G095300	*psbE-psbF-psbL*	*psbE*	Yes	Chr11:11605818-11606475
Potri.011G113600	*petB-psbH*	*psbH*	Yes	Chr11:13860093-13860825
Potri.011G113700	*psbH-psbN-psbT*	*psbN*	Yes	Chr11:13860615-13861207
Potri.013G136500	*rpl23-rpl2*	*rpl2*	Yes	Chr13:14658425-14660051
Potri.013G136600	*rpl22-rps3-rpl16*	*rpl16 & rpl22*	Yes	Chr13:14660556-14663408
Potri.013G136700	*petB-petD-rpoA*	*petB*	Yes	Chr13:14665903-14668908
Potri.013G137500	*psbT-psbN-psbH*	*psbN*	Yes	Chr13:14685919-14686512
Potri.013G138100	*psbK-psbI-trnS* ^{GCU}	*psbK*	Yes	Chr13:14723480-14724585
Potri.013G139800	*23S_rRNA-4.5S_rRNA*	**none**	Yes	Chr13:14788312-14789160
Potri.013G140300	*rps19-rpl22-rps3*	*rpl22*	Yes	Chr13:14811318-14812353
Potri.013G141000	*psbJ-psbL-psbF-psbE-petL-petG-trnW* ^{CCA} *-trnP* ^{UUG} *-psaJ*	*psbE*	Yes	Chr13: 14828217-14832398
Potri.013G142000	*trnD* ^{GUC} *-trnY* ^{GUA} *-trnE* ^{UUC} *-trnT* ^{GGU}	**none**	Yes	Chr13:14865069-14866243
Potri.013G142100	*trnC* ^{GCA} *-petN-psbM-trnD* ^{GUC}	*psbM*	Yes	Chr13:14865774-14869322
Potri.013G142400	*rpoC1-rpoC2*	*rpoC1 & rpoC2*	Yes	Chr13:14875570-14877812
Potri.013G142800	*atpA-atpF*	*atpA*	Yes	Chr13:14884552-14887338
Potri.013G143000	*trnQ* ^{UUG} *-psbK-psbI*	*psbK*	Yes	Chr13:14889638-14891106
Potri.013G143300	*rpl2-rps19-trnH* ^{GUG}	*rpl2*	Yes	Chr13:14896123-14898256
Potri.016G094100	*psaI-ycf4-cemA*	*psaI & cemA*	Yes	Chr16:8215800-8217950
Potri.019G013400	*rps19-trnH* ^{GUG} *-psbA*	*rps19*	No	Chr19:1516634-1519869
Potri.019G028300	*psbN-psbT*	*psbN*	Yes	Chr19:3197422-3198022
Potri.T103100	*trnfM* ^{CAU} *-ycf1*	*ycf1*	No	scaffold_149:61152-61778
