# Supplementary material for: Phylogenomic Analysis and Dynamic Evolution of Chloroplast Genomes in Salicaceae
Source: Front Plant Sci. 2017 Jun 20;8:1050. doi: 10.3389/fpls.2017.01050 (PMC5476734; doi:10.3389/fpls.2017.01050)
Supplement: Supplementary file 4 [file Image_1.PDF]

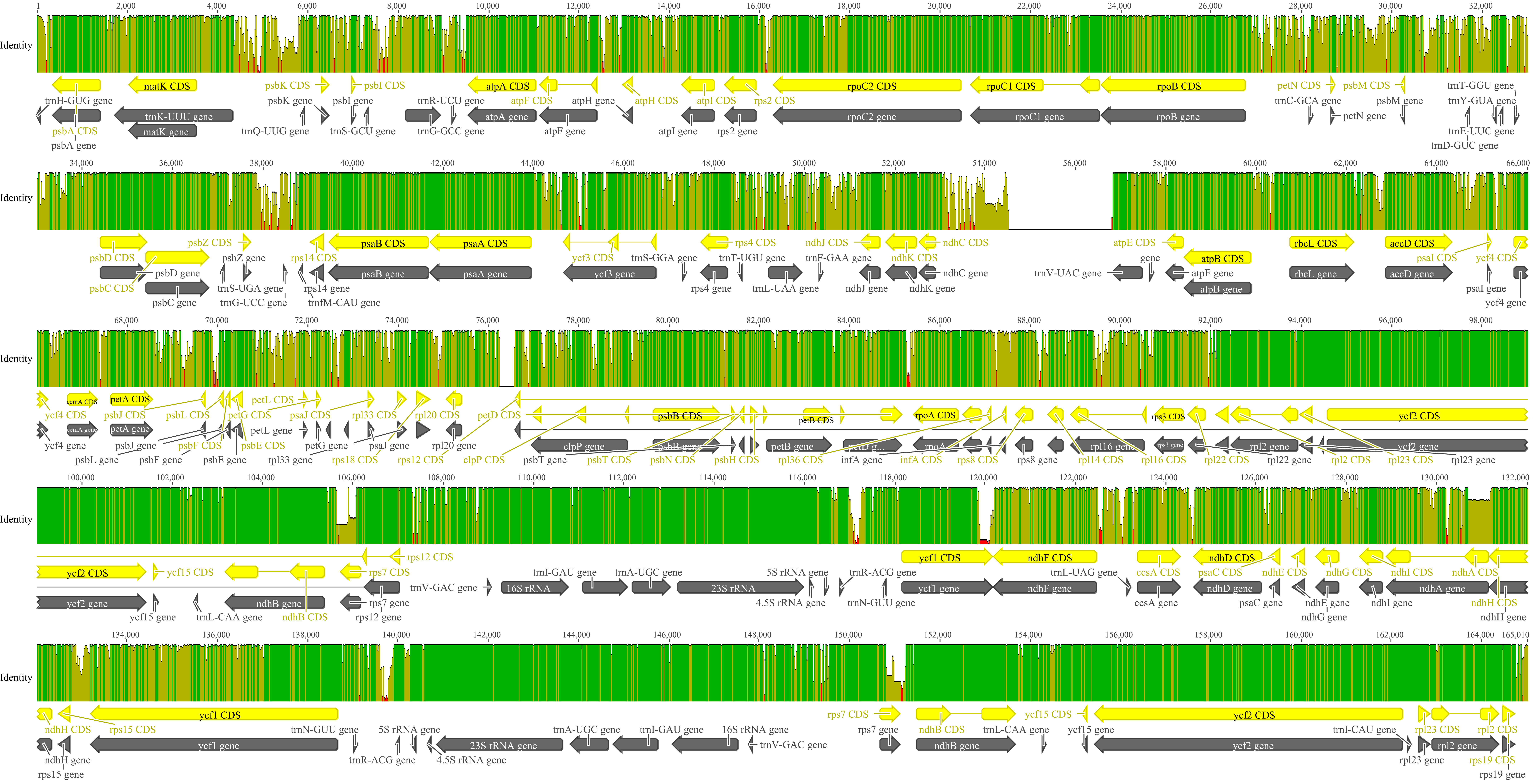

Figure S1 Visualization of alignment of the 14 Salicaceae chloroplast genome sequences.Geneious-based identity plots showing sequence identity between 3 sequenced chloroplast genomes and 11 available chloroplast genomes in GenBank, with Populus trichocarpa as a reference genome. Genome region tags are color-coded as CDS (yellow), gene (gray). Identity is displayed across all sequences for every position. Green means that the residue at the position is the same across all sequences. Yellow is for less than complete identity and red refers to very low identity for the given position.
