# Supplementary material for: Phylogenomic Analysis and Dynamic Evolution of Chloroplast Genomes in Salicaceae
Source: Front Plant Sci. 2017 Jun 20;8:1050. doi: 10.3389/fpls.2017.01050 (PMC5476734; doi:10.3389/fpls.2017.01050)
Supplement: Supplementary file 5 [file Image_2.PDF]

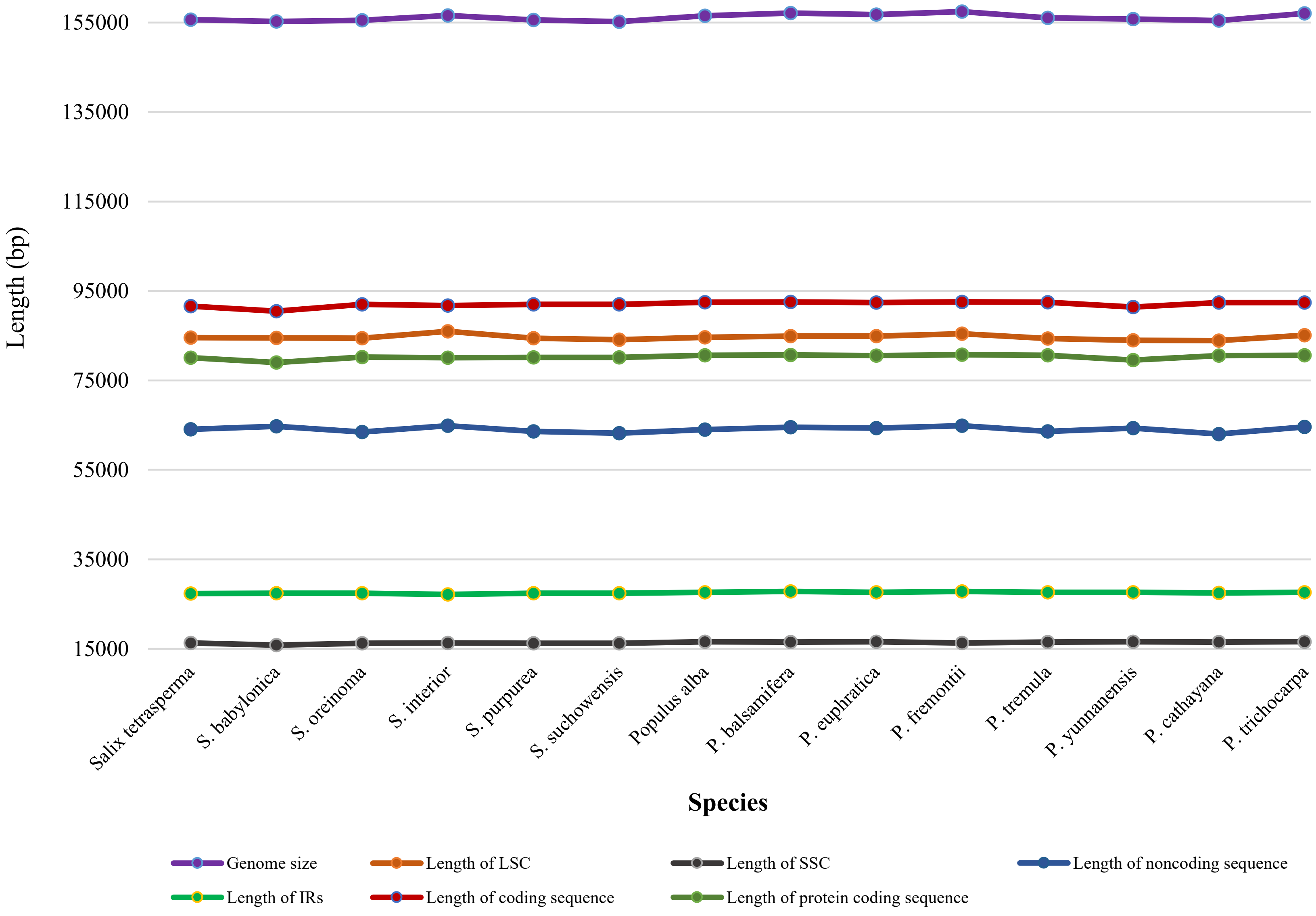

Figure S2 Illustration of length variation of chloroplast genome, LSC, SSC, IRs, coding region, protein coding region and noncoding region of analyzed Salicaceae species.
