# Supplementary material for: Phylogenomic Analysis and Dynamic Evolution of Chloroplast Genomes in Salicaceae
Source: Front Plant Sci. 2017 Jun 20;8:1050. doi: 10.3389/fpls.2017.01050 (PMC5476734; doi:10.3389/fpls.2017.01050)
Supplement: Supplementary file 6 [file Image_3.PDF]

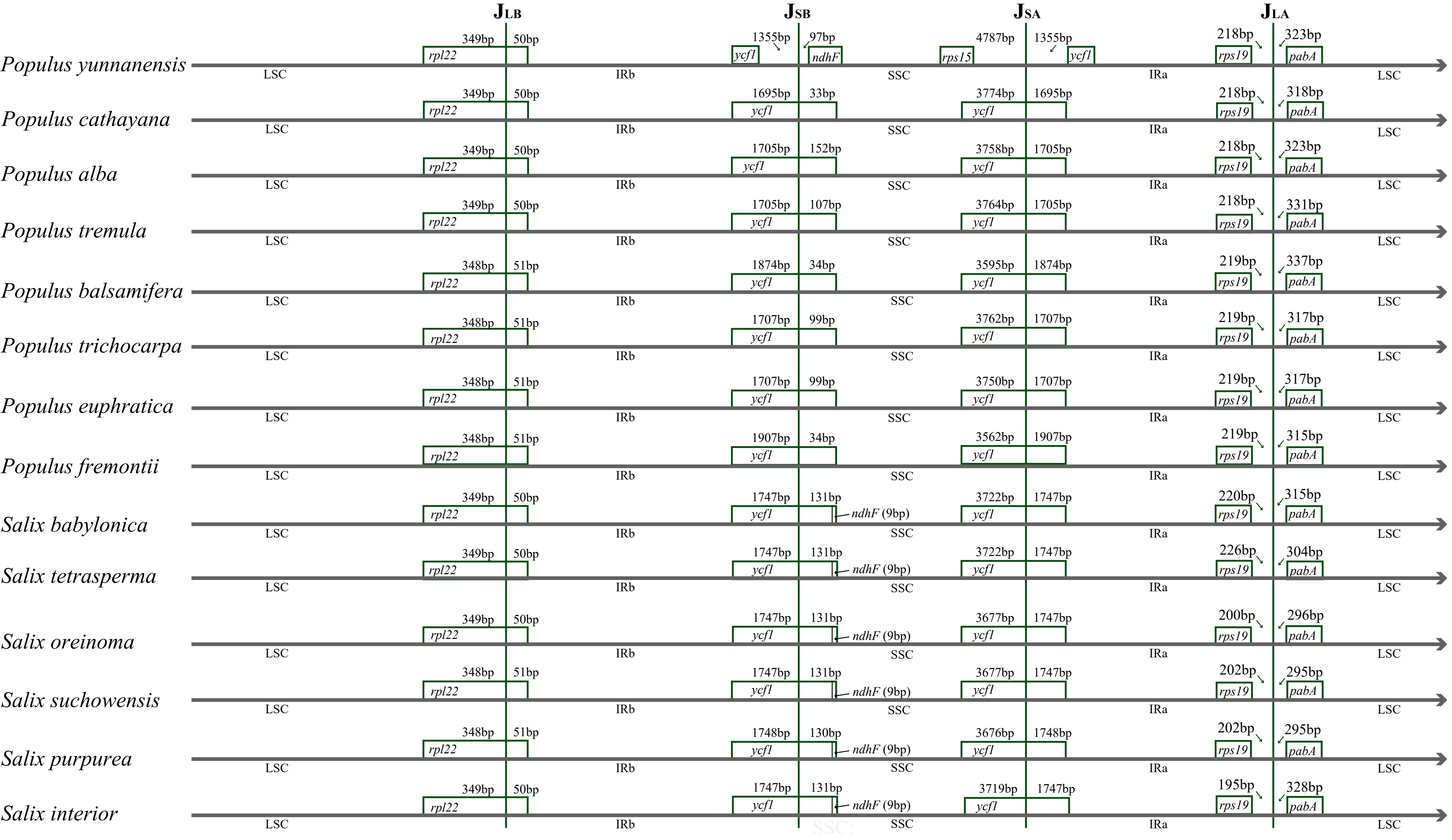

Figure S3 Comparison of IR-single copy border positions across 14 Saliaceae chloroplast genomes. The various lengths of genes (rpl22, ycf1, rps19, and psbA) adjacent to JLB, JSB, JSA, and JLA are indicated. Nine bp of ndhF is shareed with ycf1 and belong IRb.
