# Supplementary material for: Phylogenomic Analysis and Dynamic Evolution of Chloroplast Genomes in Salicaceae
Source: Front Plant Sci. 2017 Jun 20;8:1050. doi: 10.3389/fpls.2017.01050 (PMC5476734; doi:10.3389/fpls.2017.01050)
Supplement: Supplementary file 7 [file Image_4.PDF]

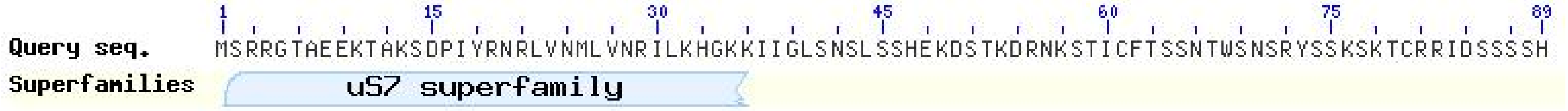

Fig. S4 Conserved domain of the shortened chloroplast *rps7* protein in *Populus cathayana* as revealed by NCBI-CDD (https://www.ncbi.nlm.nih.gov/Structure/cdd/wrpsb.cgi?) conserved domain search.
